# Risk of severe COVID-19 and mortality in patients with established chronic liver disease: a nationwide matched cohort study

**DOI:** 10.1186/s12876-021-02017-8

**Published:** 2021-11-23

**Authors:** Tracey G. Simon, Hannes Hagström, Rajani Sharma, Jonas Söderling, Bjorn Roelstraete, Emma Larsson, Jonas F. Ludvigsson

**Affiliations:** 1 Division of Gastroenterology and Hepatology, Massachusetts General Hospital, Boston, MA USA; 2 Harvard Medical School, Boston, MA USA; 3 Clinical and Translational Epidemiology Unit (CTEU), Massachusetts General Hospital, Boston, MA USA; 4 Division of Hepatology, Department of Upper GI Diseases, Karolinska University Hospital, Stockholm, Sweden; 5 Clinical Epidemiology Unit, Department of Medicine Solna, Karolinska Institutet, Stockholm, Sweden; 6 Center for Liver Disease and Transplantation, Division of Digestive and Liver Diseases, Columbia University Irving Medical Center, New York, NY USA; 7 Department of Medicine, Columbia University College of Physicians and Surgeons, New York, NY USA; 8 Department of Medical Epidemiology and Biostatistics, Karolinska Institutet, PO Box 281, 17177 Stockholm, Sweden; 9 Department of Pharmacology and Physiology, Karolinska Institutet, Stockholm, Sweden; 10 Perioperative Medicine and Intensive Care, Karolinska University Hospital, Stockholm, Sweden; 11 Department of Pediatrics, Orebro University Hospital, Orebro, Sweden; 12 Division of Epidemiology and Public Health, School of Medicine, University of Nottingham, Nottingham, UK; 13 Department of Medicine, Columbia University College of Physicians and Surgeons, New York, NY USA

**Keywords:** Corona virus, COVID, Liver disease, Cirrhosis, Survival

## Abstract

**Background and aims:**

Some, but not all, prior studies have suggested that patients with chronic liver disease are at increased risk of contracting COVID-19 and developing more severe disease. However, nationwide data are lacking from well-phenotyped cohorts with liver histology and comparisons to matched general population controls.

**Methods:**

We conducted a nationwide cohort study of all Swedish adults with chronic liver disease (CLD) confirmed by liver biopsy between 1966 and 2017 (n = 42,320), who were alive on February 1, 2020. CLD cases were matched to ≤ 5 population comparators by age, sex, calendar year and county (n = 182,147). Using Cox regression, we estimated multivariable-adjusted hazard ratios (aHRs) and 95% confidence intervals (CIs) for COVID-19 hospitalization and severe COVID-19 (intensive care admission or death due to COVID-19).

**Results:**

Between February 1 and July 31, 2020, 161 (0.38%) CLD patients and 435 (0.24%) general population controls were hospitalized with COVID-19 (aHR = 1.36, 95% CI = 1.11–1.66), while 65 (0.15%) CLD patients and 191 (0.10%) controls developed severe COVID-19 (aHR = 1.08, 95% CI = 0.79–1.48). Results were similar in patients with CLD due to alcohol use, nonalcoholic fatty liver disease, viral hepatitis, autoimmune hepatitis, and other etiologies. Among patients with cirrhosis (n = 2549), the aHRs for COVID-19 hospitalization and for severe COVID-19 were 1.08 (95% CI 0.48–2.40) and 1.23 (95% CI = 0.37–4.04), respectively, compared to controls. Moreover, among all patients diagnosed with COVID-19, the presence of underlying CLD was not associated with increased mortality (aHR = 0.85, 95% CI = 0.61–1.19).

**Conclusions:**

In this nationwide cohort, patients with CLD had a higher risk of hospitalization for COVID-19 compared to the general population, but they did not have an increased risk of developing severe COVID-19.

**Supplementary Information:**

The online version contains supplementary material available at 10.1186/s12876-021-02017-8.

## Introduction

Coronavirus disease-2019 (COVID-19), caused by infection with severe acute respiratory syndrome coronavirus-2 (SARS-CoV-2), is associated with high morbidity and mortality [1]. Established predictors of poor outcomes in patients with COVID-19 include older age, severity of the infection and clinical comorbidities, including immunosuppression and metabolic dysfunction [2]. Given the immune dysregulation that accompanies chronic liver disease (CLD), it has been hypothesized that patients with CLD may be particularly vulnerable to developing severe COVID-19. However, published data are conflicting. While several recent studies have reported significantly worse outcomes among patients with CLD or cirrhosis and COVID-19, compared to the general population or to patients with cirrhosis but without COVID-19 [3–7], others have found null associations [8]. However, those published studies have been substantially limited by the inclusion of highly-selected, hospitalized patients [3, 4, 7], or had very small sample sizes, or relied upon imprecise definitions of liver disease [5, 6]. Thus, at the population level, the natural history of SARS-CoV-2 infection in patients with underlying CLD remains largely undefined. Moreover, it is unknown whether certain etiologies of liver disease might carry a worse prognosis than others, or if the risk of developing severe COVID-19 increases in the setting of cirrhosis.

Thus, we examined the risks of hospitalization for COVID-19 and the development of severe COVID-19 (defined as ICU admission or death with confirmed COVID-19), in a population-based cohort comprised of all individuals in Sweden with biopsy-confirmed CLD. As of October 19, 2020, Sweden has recorded 103,200 COVID-19 cases and 5918 deaths, and thus has experienced higher rates of COVID-19 mortality than other neighboring countries [9]; this in turn provides a novel opportunity to study COVID-19 at the population level. Moreover, with prospectively-recorded liver histopathology data that is complete for the entire nation, this cohort permits a more comprehensive assessment of COVID-19 outcomes in patients with established CLD.

## Methods

### Study population

We conducted a nationwide, matched cohort study using the ESPRESSO (Epidemiology Strengthened by Histopathology Reports in Sweden) cohort. ESPRESSO is a nationwide cohort of prospectively-recorded liver histopathology collected from all 28 Swedish pathology departments, between 1969 and 2017 [10] covering both rural and urban areas in Sweden. Each report includes a unique, individual personal identity number (PIN), biopsy date, and Systematized Nomenclature of Medicine (SNOMED) system topography and morphology codes**.** We linked ESPRESSO to validated registers containing detailed data regarding demographics, comorbidities, prescribed medications and death. The current study was restricted to ESPRESSO participants alive on February 1, 2020.

ESPRESSO was approved by the Stockholm Ethics Board on August 27, 2014 (No. 2014/1287–31/4). Informed consent was waived as the study was register-based [11].

### Chronic liver disease (CLD) patients

CLD was defined using algorithms that we have previously described and validated in this cohort, with positive predictive values of > 90% [12, 13]. We identified all persons with an index liver biopsy between 1969 and 2017 that demonstrated viral hepatitis, nonalcoholic fatty liver disease (NAFLD), alcohol-related liver disease (ALD), autoimmune hepatitis (AIH), or another etiology of liver disease (Additional file 1: Fig. S1; Additional file 1: Table S1). Using the histopathology data, we further categorized patients with CLD according to the presence or absence of cirrhosis at the time of biopsy. We also defined individual etiologies of CLD in 5 categories (i.e. viral hepatitis, NAFLD, ALD, AIH and other CLD), using definitions outlined in Additional file 1: Table S1.

### Controls

For each CLD patient, the government agency Statistics Sweden randomly selected up to 5 general population comparators without a prior liver biopsy and CLD, according to age, sex, calendar year and county of residence (Additional file 1: Fig. S1). Comparators were derived from the Total Population Register [14], and identical exclusion criteria were applied.

### Siblings

To address potential confounding related to shared genetic or early-life factors, we also examined the risk of COVID-19 in CLD patients, compared to non-CLD full siblings. This analysis was restricted to CLD patients alive on February 1, 2020, who also had ≥ 1 sibling alive on that date. Siblings were retrieved through the Total Population Register [14].

### Covariates

In the spring of 2020, all Swedish Ethics Review Boards and government agencies were urged to facilitate COVID-19-related research. A fast track mechanism was created that allowed researchers to update existing cohorts with prospectively-collected, nationwide data regarding all deaths and hospital care for COVID-19 (through July 31, 2020), including dates of death (through July 31, 2020), to allow researchers to identify eligible subjects within their cohorts still alive in 2020. The ESPRESSO cohort was updated accordingly, but the ethics permit did not allow for updating of non-COVID-19-related information. Thus, our comorbidity data were available only through December 31, 2016.

For the current study, we used the Patient Register (hospital-based inpatient and outpatient care) to collect data regarding the following comorbidities: cardiovascular disease including thromboembolic disease, diabetes mellitus, chronic obstructive pulmonary disease (COPD), end-stage renal disease, alcohol use disorders, obesity, dyslipidemia, obstructive sleep apnea, cancer, and psychiatric disease (Additional file 1: Table S2). We also retrieved data on education level as a proxy for socioeconomic status, using the longitudinal integrated database for health insurance and labour market studies (LISA) database [15], and this was divided into four categories (i.e. missing, ≤ 9, 10–12, ≥ 13 years in full time education). Data on country of birth (Nordic vs. not Nordic country) was obtained from the Total Population Register [14].

### Outcomes

In 2020, data regarding COVID-19 intensive care unit (ICU) admissions were prospectively recorded by the Swedish intensive care registry [16], which encompasses all 83 non-neonatal ICUs in Sweden. Throughout the COVID-19 pandemic, this data recording has been mandatory for all ICUs, in cooperation with The Public Health Agency of Sweden. Information regarding COVID-19-specific deaths were obtained through the Cause of Death register [17]. We focused on two primary outcomes (Additional file 1: Table S3): (1) hospitalization with laboratory-confirmed COVID-19 (through PCR test for SARS-Cov-2) as the primary diagnosis (ICD-10: U07.1), and (2) severe COVID-19 (composite outcome, including any of the following: (a) COVID-19 ICU admission, or (b) death due to COVID-19 or (c) death within 30 days of diagnosed COVID-19 (U07.1, coded as a primary diagnosis)).

Secondary outcomes included all-cause mortality and any COVID-19 infection (a composite outcome, including any of the following: (a) clinically-diagnosed COVID-19 (i.e. ICD codes U07.1 and U07.2) in the Patient Register or in the Cause of Death register), or (b) positive record for COVID-19 from the Public Health Agency of Sweden, or (c) the development of severe COVID-19, defined above).

### Statistical analysis

Follow-up began on February 1, 2020, and continued to the first recorded date of death, COVID-19 outcome, or the end of follow-up (July 31, 2020). Our primary analyses evaluated the risk of hospitalization for COVID-19 or the development of severe COVID-19, among 42,320 patients with biopsy-confirmed CLD, compared to 182,147 matched controls. We constructed Cox proportional hazard regression models, conditioned on matching factors (i.e. age, sex, county and calendar year), to estimate multivariable-adjusted hazard ratios (aHRs) and 95% CIs. The multivariable-adjusted model further accounted for comorbidities at the index date (i.e. date of index liver biopsy confirming CLD, or the corresponding date among controls), as well as education level and country of birth.

In subgroup analyses, we examined the risk of COVID-19 hospitalization according to follow-up, sex, age at CLD diagnosis (< 18, 18 < 40, 40 < 60, ≥ 60 years), year of diagnosis (1969–89, 1990–99, 2000–09, 2010–17), country of birth, and education level. We also examined COVID-19 outcomes according to individual etiologies of CLD (i.e. NAFLD, ALD, viral hepatitis, AIH and other CLD), and according to CLD severity (defined as the presence or absence of cirrhosis). Furthermore, we explored the associations between individual covariates included in our multivariable model and the risk of either a COVID-19 hospitalization or the development of severe COVID-19.

### Sensitivity analyses

To test the robustness of our findings, we carried out a sensitivity analysis in which CLD patients were re-matched to non-CLD population controls in a 1:5 manner on December 31, 2016, using a nearest-neighbor propensity score algorithm, using a maximum caliper width of 0.2 of the pooled standard deviation of the logit of the propensity score, that included the aforementioned list of comorbidities, education level, country of birth, and exact matching variables (i.e. birth year, sex and county). This approach allowed us to more carefully account for more recent diagnoses of relevant clinical comorbidities. After applying this algorithm, > 99% of the CLD cases alive on February 1, 2020 (n = 42,008) were successfully matched to population controls at the same date.

To further address potential residual confounding, we also compared CLD patients to their full siblings. We also examined the risk of overall mortality among CLD patients and controls that were hospitalized with COVID-19, and further among CLD patients and controls with any COVID-19 diagnosis (regardless of hospitalization).

All statistical analyses were performed using SAS (version 9.4) and STATA (version 16.0). A threshold P < 0.05 was considered statistically significant.

## Results

We identified 42,320 adults with histologically-confirmed CLD between 1969–2017, who were alive on February 1, 2020, including 2549 (6.0%) with cirrhosis (Additional file 1: Fig. S1). Among those with CLD, 13,848 (32.7%) had viral hepatitis, 6350 (15.0%) had NAFLD, 2250 (5.3%) had AIH, 881 (2.1%) had ALD, and 18,991 (44.9%) had another etiology of CLD. The average age on the start date of follow-up (i.e. February 1, 2020) was 60.9 years, and 51.0% were female. Compared to population comparators, CLD patients were more likely to have underlying comorbidities, including chronic obstructive pulmonary disease (COPD), obesity/dyslipidemia, diabetes, cardiovascular disease, end-stage renal disease (ESRD) and cancer (Table 1).

**Table 1:** Baseline characteristics of patients with chronic liver disease (CLD; n = 42,320) and matched population controls (n = 182,147) on February 1, 2020

Characteristic	CLD patients (n = 42,320)	Matched controls (n = 182,147)
Females, no. (%)	21,591 (51.0%)	93,906 (51.6%)
Males, no (%)	20,729 (49.0%)	88,241 (48.4%)
Age at start of follow-up^1^		
Mean (SD)	60.9 (16.0)	59.4 (15.6)
Median (IQR)	62.8 (51.8–72.8)	61.4 (50.5–71.0)
Range, min–max	3.1–99.7	3.2–99.8
Categories, no. (%)		
< 18 years	508 (1.2%)	2479 (1.4%)
18 to < 40 years	4105 (9.7%)	19,274 (10.6%)
40 to < 60 years	13,567 (32.1%)	62,844 (34.5%)
≥ 60 years	24,140 (57.0%)	97,550 (53.6%)
Country of birth, no (%)		
Nordic country	36,036 (85.2%)	162,473 (89.2%)
Other	6284 (14.8%)	19,670 (10.8%)
Missing	0	4 (0.0%)
Level of education^3,^ no (%)		
≤ 9 years	9467 (22.4%)	33,283 (18.3%)
10–12 years	20,139 (47.6%)	83,677 (45.9%)
> 12 years	12,567 (29.7%)	64,460 (35.4%)
Missing	147 (0.3%)	727 (0.4%)
Index year^2^		
1969–1989	2966 (7.0%)	11,276 (6.2%)
1990–1999	11,722 (27.7%)	47,935 (26.3%)
2000–2009	15,979 (37.8%)	69,263 (38.0%)
2010–2017	11,653 (27.5%)	53,673 (29.5%)
Comorbidities^4^, ever before index date^2^, no. (%)		
Any cardiovascular disease	5892 (13.9%)	11,509 (6.3%)
Diabetes	2312 (5.5%)	3548 (1.9%)
Chronic obstructive pulmonary disease	513 (1.2%)	869 (0.5%)
End-stage renal disease	205 (0.5%)	118 (0.1%)
Alcohol use disorder	2450 (5.8%)	3184 (1.7%)
Obesity/dyslipidemia	2399 (5.7%)	4026 (2.2%)
Obstructive sleep apnea	492 (1.2%)	1490 (0.8%)
Cancer	5832 (13.8%)	4327 (2.4%)
Psychiatric disease	5015 (11.9%)	11,090 (6.1%)

Complete covariate data were available through December 31, 2016

CLD, chronic liver disease; no., number; SD, standard deviation; IQR, interquartile range

^1^Start date of follow-up was defined as February 1, 2020 (see “Methods”)

^2^The index date was defined as the date of liver biopsy confirming chronic liver disease (CLD), or the corresponding matching date among controls

^3^Level of education was defined in 4 categories; among subjects with missing level of education, then the highest attained education level among parents was used

^4^For definitions of comorbidities, see the Additional file 1

### Any COVID-19 hospitalization

**Fig. 1:**
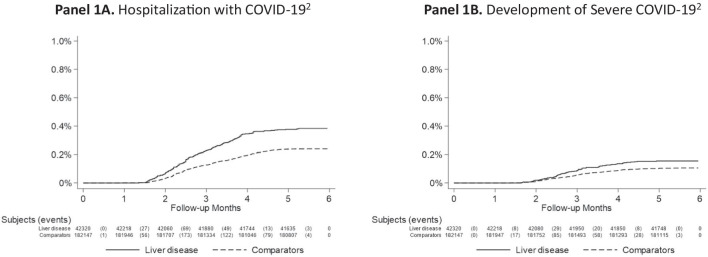
Time to COVID-19 hospitalization (panel **A**) and the development of severe COVID-19 (panel **B**), among patients with chronic liver disease (n = 42,320) and population controls (n = 182,147), matched at the index date^1^. ^1^Index date was defined as the date of index liver biopsy among patients with chronic liver disease or the equivalent matching date among population controls (see “Methods” for details). Subjects were matched by age, sex, calendar year and county of residence, and follow-up began on February 1, 2020. ^2^For definitions of COVID-19 hospitalization and development of severe COVID-19, see “Methods” and Additional file 1: Table S3

Overall, we documented 161 (0.38%) hospitalizations for COVID-19 among CLD patients (incidence rate, 7.7/1000 person-years), compared to 435 (0.24%) among controls (4.8/1000 person-years), corresponding to a multivariable aHR of 1.36 (95% CI 1.11–1.66) (Table 2; Fig. 1A). The magnitude of observed risk did not appear to increase with worsening CLD severity; specifically, compared to controls, CLD patients without cirrhosis had a multivariable aHR of 1.39 (95% CI 1.13–1.72), while those with cirrhosis had a multivariable aHR of 1.08 (95% CI 0.48–2.40).

**Table 2:** Risk of COVID-19 hospitalization and severe COVID-19 in patients with chronic liver disease^1^ (n = 42,320) and matched population controls (n = 182,147), from February 1 to July 31, 2020

Outcome	N events (%)		Time at risk (years)		Incidence rate (95% CI) per 1000 PY		HR^2^ (95% CI)	Adjusted HR^3^ (95% CI)
	Liver disease	Comparators	Liver disease	Comparators	Liver disease	Comparators		
**Main outcomes**								
Hospital admission	161 (0.38%)	435 (0.24%)	20,786	89,945	7.7 (6.5–8.9)	4.8 (4.4–5.3)	1.49 (1.24–1.79)	1.36 (1.11–1.66)
Severe COVID-19^1^	65 (0.15%)	191 (0.10%)	20,816	90,021	3.1 (2.4–3.9)	2.1 (1.8–2.4)	1.16 (0.87–1.55)	1.08 (0.79–1.48)
**Secondary outcomes**								
Main outcomes combined	182 (0.43%)	504 (0.28%)	20,786	89,945	8.8 (7.5–10.0)	5.6 (5.1–6.1)	1.39 (1.17–1.65)	1.28 (1.06–1.54)
All-cause mortality	622 (1.47%)	1115 (0.61%)	20,822	90,034	29.9 (27.5–32.2)	12.4 (11.7–13.1)	2.00 (1.81–2.21)	1.62 (1.45–1.81)
Any COVID-19	566 (1.34%)	1832 (1.01%)	20,711	89,698	27.3 (25.1–29.6)	20.4 (19.5–21.4)	1.29 (1.17–1.42)	1.16 (1.05–1.28)

N., number; CI, confidence interval; PY, person-years; HR, hazard ratio

^1^Severe COVID-19 was a composite outcome, defined by any of the following: (a) COVID-19 ICU admission, or (b) death due to COVID-19 or (c) death within 30 days of diagnosed COVID-19 (for details, see “Methods”)

^2^Conditioned on matching set (age, sex, county, and calendar period)

^3^Conditioned on matching set and further adjusted for education, Nordic country of birth, and medical comorbidities at the index date (i.e. the date of index liver biopsy or corresponding matching date among comparators), including: cardiovascular disease, diabetes, chronic obstructive pulmonary disease, end-stage renal disease, alcohol use disorder, obesity/dyslipidemia, obstructive sleep apnea, cancer and psychiatric disease

No individual etiology of CLD was associated with significantly increased risk of COVID-19 hospitalization. Compared to controls, we observed null associations for viral hepatitis (aHR = 1.12, 95% CI = 0.78–1.62), NAFLD (aHR = 1.50, 95% CI = 0.91–2.48), ALD (aHR = 0.70, 95% CI = 0.19–2.62), AIH (aHR = 1.54, 95% CI = 0.56–4.27) or other etiologies of liver disease (aHR = 1.30, 95% CI = 0.93–1.81). Furthermore, the HRs for COVID-19 hospitalization did not differ substantially by sex, calendar year of CLD diagnosis, country of birth or education level (Table 3). However, the risk of COVID-19 hospitalization did vary substantially according to age at the index biopsy (P-interaction = < 0.001), and according to age at the start date of follow-up (P-interaction 0.01; Table 3).

**Table 3:** Risk of COVID-19 Hospitalization Overall and by Subgroups in Patients with Chronic Liver Disease (n = 42,320) and Matched Population Controls (n = 182,147) from February 1 to July 31, 2020

Group	N (%)		N events (%)		Incidence rate (95% CI) per 1000 PY		HR* (95% CI)	Adjusted HR** (95% CI)
	Liver disease	Comparators	Liver disease	Comparators	Liver disease	Comparators		
**Overall**	42 320 (100%)	182 147 (100%)	161 (0.38%)	435 (0.24%)	7.7 (6.5–8.9)	4.8 (4.4–5.3)	1.49 (1.24–1.79)	1.36 (1.11–1.66)
Follow-up, months								
0 to < 1	42 320 (100%)	182 147 (100%)	– (0.00%)	1 (0.00%)	–	0.1 (0.0–0.2)	–	–
1 to < 2	42 218 (99.8%)	181 946 (99.9%)	27 (0.06%)	56 (0.03%)	7.7 (4.8–10.6)	3.7 (2.7–4.7)	1.91 (1.20–3.04)	1.42 (0.83–2.43)
2 to < 3	42 060 (99.4%)	181 707 (99.8%)	69 (0.16%)	173 (0.10%)	19.7 (15.1–24.4)	11.4 (9.7–13.1)	1.58 (1.19–2.10)	1.61 (1.17–2.22)
3 to < 4	41 880 (99.0%)	181 334 (99.6%)	49 (0.12%)	122 (0.07%)	14.1 (10.1–18.0)	8.1 (6.6–9.5)	1.60 (1.15–2.25)	1.56 (1.07–2.26)
4 to < 5	41 744 (98.6%)	181 046 (99.4%)	13 (0.03%)	79 (0.04%)	3.7 (1.7–5.8)	5.2 (4.1–6.4)	0.71 (0.39–1.28)	0.45 (0.22–0.92)
5 to < 6	41 635 (98.4%)	180 807 (99.3%)	3 (0.01%)	4 (0.00%)	0.9 (0.0–1.9)	0.3 (0.0–0.6)	2.78 (0.60–12.78)	–
Sex								
Females	21 591 (51.0%)	93 906 (51.6%)	60 (0.28%)	195 (0.21%)	5.7 (4.2–7.1)	4.2 (3.6–4.8)	1.24 (0.92–1.65)	1.03 (0.74–1.43)^Ψ^
Males	20 729 (49.0%)	88 241 (48.4%)	101 (0.49%)	240 (0.27%)	9.9 (8.0–11.9)	5.5 (4.8–6.2)	1.69 (1.34–2.14)	1.64 (1.26–2.15)
Age at index date								
< 18 years	2 769 (6.5%)	12 877 (7.1%)	2 (0.07%)	5 (0.04%)	1.5 (0.0–3.5)	0.8 (0.1–1.5)	1.93 (0.37–9.98)	2.43 (0.35–16.95)^Ψ^
18 to < 40 years	13 574 (32.1%)	61 290 (33.6%)	38 (0.28%)	90 (0.15%)	5.7 (3.9–7.5)	3.0 (2.4–3.6)	1.86 (1.27–2.73)	1.80 (1.18–2.77)
40 to < 60 years	18 446 (43.6%)	79 416 (43.6%)	85 (0.46%)	206 (0.26%)	9.4 (7.4–11.4)	5.3 (4.5–6.0)	1.72 (1.33–2.23)	1.64 (1.24–2.18)
≥ 60 years	7 531 (17.8%)	28 564 (15.7%)	36 (0.48%)	134 (0.47%)	9.9 (6.6–13.1)	9.6 (8.0–11.2)	0.91 (0.62–1.32)	0.69 (0.45–1.07)
Index year								
1969–1989	2 966 (7.0%)	11 276 (6.2%)	8 (0.27%)	20 (0.18%)	5.5 (1.7–9.3)	3.6 (2.0–5.2)	1.45 (0.63–3.32)	0.98 (0.36–2.69)
1990–1999	11 722 (27.7%)	47 935 (26.3%)	50 (0.43%)	137 (0.29%)	8.7 (6.3–11.1)	5.8 (4.8–6.8)	1.38 (1.00–1.92)	1.44 (1.02–2.04)
2000–2009	15 979 (37.8%)	69 263 (38.0%)	56 (0.35%)	165 (0.24%)	7.1 (5.3–9.0)	4.8 (4.1–5.6)	1.35 (0.99–1.84)	1.07 (0.75–1.53)
2010–2017	11 653 (27.5%)	53 673 (29.5%)	47 (0.40%)	113 (0.21%)	8.2 (5.9–10.6)	4.3 (3.5–5.0)	1.84 (1.31–2.60)	1.63 (1.08–2.45)
Age at start of follow-up								
< 18 years	508 (1.2%)	2 479 (1.4%)	– (0.00%)	– (0.00%)	–	–	–	–
18 to < 40 years	4 105 (9.7%)	19 274 (10.6%)	7 (0.17%)	7 (0.04%)	3.4 (0.9–6.0)	0.7 (0.2–1.3)	4.67 (1.63–13.36)	11.45 (2.17–60.51)^Ψ^
40 to < 60 years	13 567 (32.1%)	62 844 (34.5%)	48 (0.35%)	95 (0.15%)	7.2 (5.1–9.2)	3.1 (2.4–3.7)	2.44 (1.72–3.45)	2.02 (1.34–3.05)
≥ 60 years	24 140 (57.0%)	97 550 (53.6%)	106 (0.44%)	333 (0.34%)	9.0 (7.3–10.7)	6.9 (6.2–7.7)	1.21 (0.97–1.51)	1.12 (0.88–1.43)
Country of birth								
Nordic	36 036 (85.2%)	162 473 (89.2%)	115 (0.32%)	304 (0.19%)	6.5 (5.3–7.7)	3.8 (3.4–4.2)	1.46 (1.17–1.82)	1.36 (1.07–1.72)
Other	6 284 (14.8%)	19 670 (10.8%)	46 (0.73%)	131 (0.67%)	14.9 (10.6–19.2)	13.5 (11.2–15.8)	1.87 (1.00–3.49)	1.73 (0.83–3.60)
Level of education								
≤ 9 years	9 467 (22.4%)	33 283 (18.3%)	50 (0.53%)	119 (0.36%)	10.8 (7.8–13.8)	7.3 (6.0–8.6)	1.73 (1.05–2.87)	1.45 (0.83–2.53)
10–12 years	20 139 (47.6%)	83 677 (45.9%)	76 (0.38%)	196 (0.23%)	7.7 (6.0–9.4)	4.7 (4.1–5.4)	1.44 (1.05–1.99)	1.74 (1.20–2.51)
> 12 years	12 567 (29.7%)	64 460 (35.4%)	34 (0.27%)	113 (0.18%)	5.5 (3.6–7.3)	3.5 (2.9–4.2)	1.06 (0.63–1.80)	1.05 (0.58–1.92)

N, number; CI, confidence interval; PY, person-years; HR, hazard ratio

*Conditioned on matching set (age, sex, county, and calendar period)

**Conditioned on matching set and further adjusted for education, Nordic country of birth, and medical comorbidities at index date (cardiovascular disease, diabetes, chronic obstructive pulmonary disease, end-stage renal disease, alcohol use disorder, obesity/dyslipidemia, obstructive sleep apnea, cancer, psychiatric disease)

^Ψ^P-interaction values for sex, age at the index date and age at the start of follow-up = 0.06, < 0.001 and 0.01, respectively

### Severe COVID-19

We documented a total of 65 (0.15%) severe COVID-19 infections (i.e. intensive care admission or death) among CLD patients (incidence rate, 3.1/1000 person-years), compared to 191 (0.10%) among population controls (incidence rate, 2.1/1000 person-years); this corresponded to an aHR of 1.08 (95% CI 0.79–1.48) (Table 2; Fig. 1B). The HRs for developing severe COVID-19 compared to non-CLD controls were significantly higher among patients aged 40- < 60 years at the start of follow-up (aHR 2.20, 95% CI 0.67–7.28), compared to those ≥ 60 years of age (aHR 0.97, 95% CI 0.69–1.37)(P-interaction = 0.02), but otherwise they did not differ substantially by sex, calendar year of CLD diagnosis, country of birth or education level (Additional file 1: Table S4). These estimates were also consistent in patients without cirrhosis (aHR = 1.05, 95% CI = 0.75–1.47), or with cirrhosis (aHR = 1.23, 95% CI = 0.37–4.04), and further among CLD patients with underlying viral hepatitis (aHR = 0.85, 95% CI = 0.40–1.79), NAFLD (aHR = 0.96, 95% CI = 0.42–2.21), AIH (aHR = 0.23, 95% CI = 0.03–1.78), and other etiologies of liver disease (aHR = 1.03, 95% CI = 0.64–1.65). There were only 5 recorded cases of severe COVID-19 among patients with ALD, thus aHRs were not calculated.

Compared to controls, CLD patients had increased risk of all-cause mortality during the study period (aHR = 1.62, 95% CI = 1.45–1.81). They were also at a 16% increased risk of any COVID-19 infection (i.e. any clinically-diagnosed COVID-19 or a confirmed positive record of COVID-19, or severe COVID-19; aHR = 1.16, 95% CI = 1.05–1.28) (Table 2).

We also examined the relationships between individual covariates included in our multivariable model and the risk of COVID-19 hospitalization or the development of severe COVID-19. As shown in Additional file 1: Table S5, patients with diabetes and obesity or dyslipidemia had significantly higher risk of developing severe COVID-19, compared to individuals without those comordibities, which is consistent with prior literature.

### Sensitivity analyses

To address underlying comorbidities relevant to COVID-19 outcomes, we conducted a propensity score-matched sensitivity analysis (Additional file 1: Fig. S2). After propensity score-matching, comorbidities were well-balanced between CLD cases (n = 42,008) and population controls (n = 208,004), including diabetes, obesity/dyslipidemia, cardiovascular disease, ESRD, COPD and obstructive sleep apnea (Additional file 1: Table S6). Compared to propensity matched controls, CLD patients had a modestly increased risk of COVID-19 hospitalization (aHR = 1.20, 95% CI = 1.01–1.43), but no significantly increased risk of developing severe COVID-19 (aHR = 1.16, 95% CI = 0.88–1.52) (Additional file 1: Table S7; Additional file 1: Fig. S3). Consistent with our primary analyses, the HRs for both COVID-19 hospitalization and for developing severe COVID-19 did not differ substantially by sex, age at the index biopsy, calendar year of CLD diagnosis, country of birth, education level or underlying cirrhosis (Additional file 1: Tables S8A, S8B). Results also remained similarly null in subgroups of CLD patients with viral hepatitis, NAFLD, ALD, AIH, and other etiologies of CLD (data not shown).

After matching CLD patients to full sibling comparators (Additional file 1: Table S9), we observed similar results (aHRs for COVID-19 hospitalization and for severe COVID-19 were, 1.42 (95% CI = 0.99–2.03); and 1.09 (95% CI = 0.55–2.16), respectively), and none of these risk estimates achieved statistical significance (Additional file 1: Table S10; Additional file 1: Fig. S4).

**Fig. 2:**
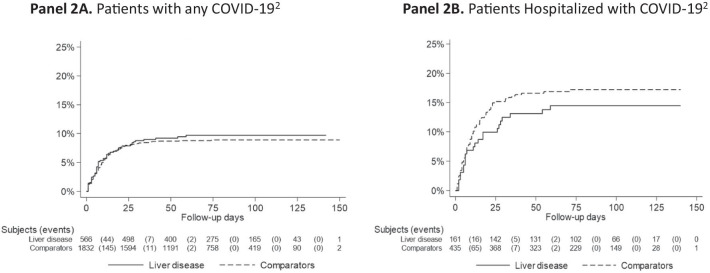
Time from any COVID-19 infection (panel **A**) or hospitalization with COVID-19 (panel **B**) to all-cause mortality, among patients with chronic liver disease and population controls, matched at the index date^1^. ^1^Index date was defined as the date of index liver biopsy among patients with chronic liver disease or the equivalent matching date among population controls (see “Methods” for details). Subjects were matched by age, sex, calendar year and county of residence, and follow-up began on February 1, 2020. ^2^For definitions of any COVID-19 and hospitalization with COVID-19, see “Methods” and Additional file 1: Table S3

In further analyses, we restricted our sample to (1) CLD patients and controls diagnosed with any COVID-19 (i.e. patients either tested positive for COVID-19 or were hospitalized with COVID-19), or (2) CLD patients and controls hospitalized with COVID-19 (Additional file 1: Table S3). The presence of underlying CLD was not significantly associated with excess mortality among patients with any COVID-19 (aHR = 0.85, 95% CI = 0.61–1.19), or among patients hospitalized with COVID-19 (aHR = 0.91, 95% CI = 0.54–1.51) (Table 4; Fig. 2). Importantly, results were similar among patients with any COVID-19 but no cirrhosis (aHR = 0.80, 95% CI 0.56–1.15) and among those with any COVID-19 and with cirrhosis (aHR = 0.81, 95% CI = 0.30–2.17), compared to matched controls (Table 4). We also observed similar null associations after CLD patients and controls were propensity score-matched (Additional file 1: Fig. S5).

**Table 4:** Risk of all-cause mortality in CLD patients with COVID-19^1^ and population controls with COVID-19, matched at the index date

All-Cause Mortality	N exposed (%)		N death (%)		Incidence rate (95% CI)		HR (95% CI)	Adjusted HR* (95% CI)
	Liver disease	Comparators	Liver disease	Comparators	Liver disease	Comparators		
Any COVID-19 infection^1^	566	1832	53 (9.4%)	158 (8.6%)	0.5 (0.3–0.6)	0.5 (0.4–0.5)	1.08 (0.79–1.48)	0.85 (0.61–1.19)
Cirrhosis	51	114	9 (17.6%)	15 (13.2%)	1.0 (0.3–1.6)	0.7 (0.4–1.1)	1.36 (0.59–3.10)	0.81 (0.30–2.17)
No Cirrhosis	515	1718	44 (8.5%)	143 (8.3%)	0.4 (0.3–0.6)	0.5 (0.4–0.5)	1.02 (0.73–1.43)	0.80 (0.56–1.15)
Hospitalized with COVID-19	161	435	23 (14.3%)	74 (17.0%)	0.6 (0.4–0.9)	0.8 (0.6–1.0)	0.82 (0.51–1.31)	0.91 (0.54–1.51)

CLD, chronic liver disease; N, number; CI, confidence interval; PY, person-years; HR, hazard ratio

^*^Adjusted for age, sex, index year, education, Nordic country of birth, and medical comorbidities at index date (i.e. cardiovascular disease, diabetes, chronic obstructive pulmonary disease, end-stage renal disease, alcohol use disorder, obesity/dyslipidemia, obstructive sleep apnea, cancer, psychiatric disease)

^1^Any COVID-19 was defined as either a recorded diagnosis of COVID-19 or hospitalization for COVID-19 (see “Methods” and Additional file 1: Table S3 for definitions)

## Discussion

In this nationwide, population-based cohort of 42,320 patients with biopsy-confirmed CLD, we found a modestly increased risk of COVID-19 hospitalization, but no increased risk of developing severe COVID-19, compared to matched population controls. These findings were similar across all etiologies of CLD, and they were consistent in subjects with and without underlying cirrhosis. They also were robust in numerous sensitivity analyses, including after propensity score-matching for important clinical comorbidities, and further after comparing CLD patients with full siblings. Perhaps most importantly of all, among individuals diagnosed with verified COVID-19, the presence of CLD—including underlying cirrhosis—did not predict overall mortality.

Published data linking CLD to COVID-19 outcomes are limited and conflicting, with some studies demonstrating higher rates of ICU admissions or death in CLD patients with COVID-19 [3–7], while others have found null associations [8]. Our findings are broadly consistent with a recent meta-analysis of 3 studies, which found that the odds of developing severe COVID-19 did not differ substantially, between patients with pre-existing CLD and controls (pooled OR = 0.81, 95% CI 0.31–2.09) [8]. However, that meta-analysis included just 70 patients with CLD, and recorded very few events, and thus had limited generalizability and produced imprecise risk estimates. Moreover, prior registry-based studies to date have restricted enrollment to subjects admitted to the hospital or to the ICU with COVID-19, which could introduce substantial selection bias, as patients were identified using non-systematic approaches that focused uniquely on severe cases or those with active Hepatology involvement in patient care [4, 18].

In contrast, by leveraging the comprehensive, nationwide data of the Swedish healthcare registers, the current study included more subjects with confirmed CLD than any prior study, to date, as well as over 182,000 matched population controls. This allowed us to calculate more precise absolute and relative risks for the development of clinically-meaningful COVID-19, on a nationwide scale.

We found that patients with biopsy-confirmed CLD had an increased risk of hospitalization for COVID-19, but the magnitude of that risk was modest. It is possible that patients with established CLD were more likely to receive COVID-19 testing, and further that there may have been a lower threshold to hospitalize this vulnerable population, upon receipt of a positive COVID-19 test. In contrast, the rates of developing severe COVID-19 were similar when we compared CLD patients to matched population controls; moreover, among patients diagnosed with COVID-19, the presence of underlying CLD did not predict overall mortality. These latter findings are less susceptible to ascertainment bias, and they remained similar in CLD patients with and without cirrhosis. Overall, our results are quite reassuring, for they suggest that patients with biopsy-confirmed CLD—including those with cirrhosis—are not predisposed to developing severe COVID-19.

This study benefits from a nationwide population with prospectively-recorded histopathology and strict, validated definitions of specific etiologies of CLD and cirrhosis. We leveraged comprehensive data from all of Sweden’s 83 non-neonatal intensive care units, and the Swedish registers provide near-complete follow-up for the entire population, permitting calculation of more precise absolute and relative risk estimates, while minimizing the inherent limitations of previous, smaller studies. We constructed a propensity score-matched model to account for comorbidities and relevant medication use recorded up to 2016, and our unique sibling analyses enabled us to minimize potential confounding from shared genetics and early environmental factors.

We acknowledge several limitations. First, CLD was defined histologically and not all patients with CLD (including some with decompensated cirrhosis) undergo biopsy, which could introduce selection bias. Due to our study design, we may have had a lower prevalence of patients with advanced cirrhosis, because such patients would be more likely to have died between the date of index liver biopsy and the start date of the study period. We cannot rule out that an overrepresentation of patients with milder cirrhosis contributed to the lack of association between cirrhosis and severe COVID-19 in our paper. That our study design requested patients to survive from 2016 to 2020 is also likely to explain the low prevalence of persons with ALD, since such patients also have a dismal prognosis [19]. Thus, our results should be interpreted carefully when applied to patients with decompensated cirrhosis that might be at a higher risk for severe outcomes. However, our hazard estimates were consistent in subgroups restricted to more recent time-periods, and they also are broadly consistent with those reported in a recent meta-analysis, which found a null association between underlying CLD and the odds of developing severe COVID-19 (n = 70 subjects with CLD from 3 studies; pooled OR, 0.81, 95% CI 0.31–2.09) [8]. Second, ICU admission depends upon numerous factors including age and comorbidities, and we lacked ICU-level data regarding thresholds for admission. We cannot rule out that admission criteria to the ICU may have changed during the pandemic with a secondary impact on mortality rates. While we were unable to examine the risk of invasive ventilation, such measures are only offered through the ICU in Sweden. Third, while it is reassuring that severe COVID-19 did not appear to occur more frequently in CLD patients, compared to controls, with a relatively small number of endpoints it is possible that our statistical power was limited in certain subgroups and even for one of our main outcomes: severe COVID-19.

Fourth, despite multivariable adjustment and propensity-score matching for numerous comorbidities including COPD, obesity/dyslipidemia, cardiovascular disease, ESRD and alcohol use disorder, our cohort lacked detailed data regarding body mass index or smoking, which may be important risk factors for developing severe COVID-19. We also lacked access to clinical data to calculate Child–Pugh or model for end-stage liver disease (MELD) scores in persons with cirrhosis. Moreover, we did not have data on progression, or regression, of liver disease or covariates after December 31, 2016, nor did we have data regarding treatment for viral hepatitis or autoimmune liver disease. Therefore, some cases might have progressed to cirrhosis or had a modest degree of regression, after the initial biopsy. It is also possible that a small number of controls may have developed CLD in 2017–2020, but such numbers should be small and unlikely to impact on our risk estimates. Fifth, the Swedish population is primarily Caucasian, underscoring the need for continued research in racially and ethnically diverse populations. Finally, by nature of the national registries, it is possible that some patients with asymptomatic COVID-19 were not included as cases. However, the outcomes assessed in this study are likely to be more informative and clinically relevant for patients and providers, as they capture more severe manifestations of disease. We also had no individual-level information regarding specific precautions taken by patients with and without CLD, during the pandemic.

## Clinical implications

The lack of a clear association between CLD and severe COVID-19 differentiates liver disease from for instance cardiovascular disease which has been linked to a poorer prognosis in COVID-19. This also points towards the specificity of COVID-19 and it should be emphasized that CLD may very well be an important risk factor for both incident and death from other pandemics in the future. We also acknowledge that our study design requiring patients to survive from 2016 to 2020 may have impacted on our risk estimates.

In conclusion, within a nationwide, population-based cohort of more than 42,000 patients with biopsy-confirmed CLD, we found a modestly increased risk of COVID-19 hospitalization, but no increased risk of developing severe COVID-19. Moreover, among individuals diagnosed with COVID-19, the presence of underlying CLD did not predict overall mortality.

## Supplementary Information

**Additional file 1:** Definitions of chronic liver disease, baseline medical comorbidities, COVID-19 outcomes, study participants and risk estimates for COVID-19 in chronic liver disease.

## Data Availability

The data that support the findings of this study are available from the 28 Swedish Pathology Departments and from the government administered LISA database but restrictions apply to the availability of these data, which were used under license for the current study, and so are not publicly available. Readers should contact the two government agencies: *Swedish National Board of Health and Welfare*, and *Statistics Sweden*, to access these data.

## Abbreviations

aHR: Adjusted hazard ratio
AIH: Autoimmune hepatitis
ALD: Alcohol-related liver disease
CI: Confidence interval
CLD: Chronic liver disease
COPD: Chronic obstructive pulmonary disease
COVID-19: Coronavirus disease-2019 (COVID-19)
ESPRESSO: Epidemiology Strengthened by Histopathology Reports in Sweden
ESRD: End-stage renal disease
ICD: International classification of diseases
ICU: Intensive care unit
NAFLD: Nonalcoholic fatty liver disease
PIN: Personal identity number (PIN)
SARS-CoV-2: Severe acute respiratory syndrome coronavirus-2 (SARS-CoV-2)
SNOMED: Systematized Nomenclature of Medicine
