# Supplementary material for: Risk of severe COVID-19 and mortality in patients with established chronic liver disease: a nationwide matched cohort study
Source: BMC Gastroenterol. 2021 Nov 23;21:439. doi: 10.1186/s12876-021-02017-8 (PMC8609512; doi:10.1186/s12876-021-02017-8)

**SUPPLEMENTARY APPENDIX**

**Table S1** Definitions of Chronic Liver Disease using SNOMED and ICD codes

| **Disease/Condition** | **Topographic code** | **SNOMED codes** | **ICD-codes** | | | **Exclusion criteria** |
| --- | --- | --- | --- | --- | --- | --- |
|  |  |  | **ICD-8** | **ICD-9** | **ICD-10** |  |
| Any liver disease | T56 |  |  |  |  |  |
|  |  |  |  |  |  |  |
| **Severity of liver disease** |  |  |  |  |  |  |
| No cirrhosis | T56 |  |  |  |  | Exclude any cirrhosis |
| Compensated cirrhosis | T56 | M495 |  |  |  | Exclude decompensated cirrhosis/liver failure |
| Decompensated  cirrhosis/liver failure | T56 | M495 | 570;  456; 785,3 | 570,  456A, 456B, 456C, 789F, 572E | K72.0,  I85.0, I85.9, K76.7, R18.9 |  |
| **Etiology of liver disease** |  |  |  |  |  |  |
| Viral hepatitis | T56 | D052 | 070; 999,20 | 070 | B15, B16, B17, B18, B19,  B008, B251 |  |
| Alcohol-related liver disease (ALD) | T56 |  | 571,00; 571,01 | 571A, 571B, 571C, 571D | K70 | Exclude: Viral hepatitis |
| Autoimmune hepatitis (AIH) | T56 |  | 573,0; 571,9 | 571E, 573D | K75.4 | Exclude: Viral hepatitis, ALD |
| Nonalcoholic Fatty Liver Disease (NAFLD) | T56 | M5008, M5520 |  |  |  | Exclude: Viral hepatitis, ALD, AIH |
| Other | T56 |  |  |  |  | Exclude: Viral hepatitis, ALD, AIH, NAFLD |

**Table S2** Definitions of liver transplantation and baseline medical comorbidities ever before December 31, 2016

| **Comorbidity** | **ICD10**  **(1997-)** | **ICD9**  **(1987-1996)** | **ICD8**  **(1969-1986)** | **Procedure codes** |
| --- | --- | --- | --- | --- |
|  |  |  |  |  |
| Liver transplantation | Z94.4 | V42H |  | ≥1997: JJC  <1997: 5200-5299 |
| Cardiovascular disease |  |  |  |  |
| - Hypertension | I10-I15 | 401-405 | 400-404 |  |
| - Ischemic heart disease | I20-I25 | 410-414 | 410-414 |  |
| - Thromboembolic disease | I26, I80-I82 | 451-453, 415B | 450-453 |  |
| - Deep venous thrombosis | I80.1-I80.2, I81, I82.2-I82.9 | 451B, 452, 453C, 453D, 453W, 453X | 451-452 |  |
| - Cerebrovascular disease | I60-I69 | 430-434, 436-438 | 430-434, 436-438 |  |
| - Congestive heart failure | I50 | 428 | 427,0-427,1, 428,9 |  |
|  |  |  |  |  |
| Diabetes | E10-E14, O24 | 250, 648A | 250 |  |
| Chronic obstructive pulmonary disease | J41-J44 | 491-492, 496 | 490; 491,01-491,02; 491,04; 492 |  |
| End-stage renal disease | N18.0, N18.5, Z49, Z99.2, Z94.0 | 585, V45B, V56, V42A | Y29,01 | 9200; V9200; 9212; V9212; 9314; V9531; DR012; DR013; DR016; DR024; QF006; 9211; V9211; 9213; V9213; V9532; DR015; DR023; DR055; DV056; 9219; V9219; 9223; V9223; DR017; DR020; DR055; DR056; 6070; KAS10; KAS20 |
| Alcohol-related liver disease | K70 | 571A, 571B, 571C, 571D | 571,00; 571,01 |  |
| Alcohol use disorder | F10, E24.4, F04.9, G31.2, G62.1, G72.1, I42.6, K29.2, K85.2, K86.0, O35.4, X65, Y15, Y91 | 255, 294A, 291, 303, 305A, 357F, 425F, 535D, 655E, 980 | 571,09; 258, 291,1; 299 |  |
| Obesity / Dyslipidemia | E78, E65, E66 | 272, 278 | 272,00-272,01  277,99 |  |
| Obstructive sleep apnea | G47.3 | - | - |  |
| Cancer | C00-C97 (not C44) | 140-208 | 140-209 (not 173, 175-179 or 208) |  |
| Psychiatric disease | F06-F09, F20-F99 (not F55.9) | 290-307 (not 303-305, or 307F) | 290-301 |  |

**Table S3** Definitions of COVID-19 Outcomes

| **Outcome** | **Description** | **ICD10**  **(1997-)** |
| --- | --- | --- |
|  |  |  |
| **Primary outcomes** |  |  |
| Hospitalization with COVID-19 | Hospital admission with laboratory-confirmed COVID-19 as the primary diagnosis | U07.1 |
| Severe COVID-19 | Composite outcome of:  a) COVID-19 intensive care admission, or  b) death due to COVID-19 as the underlying cause of death, or  c) death within 30 days of diagnosed COVID-19 as the primary diagnosis in the Patient register | U07.1 |
|  |  |  |
| **Secondary outcomes** |  |  |
| Main outcomes combined | The definitions for main outcomes combined | U07.1 |
| All-cause mortality | All-cause mortality | - |
| Any COVID-19 | Composite outcome of:  a) ICD codes U07.1 and U07.2 in the Patient Register, or  b) ICD codes U07.1 and U07.2 in the Cause of Death Register, or  c) a positive record for COVID-19 from the Swedish Public Health Agency, or  d) COVID-19 intensive care | U07.1, U07.2 |

**Table S4.** Risk of Severe COVID-19 Overall and by Subgroups in Patients with Chronic Liver Disease (n=42,320) and Matched Population Controls (n=182,147) from February 1 to July 31, 2020

| **Group** | **N (%)** | | **N events (%)** | | **Incidence rate (95% CI)**  **per 1000 PY** | | **HR***  **(95%CI)** | **Adjusted HR****  **(95%CI)** |
| --- | --- | --- | --- | --- | --- | --- | --- | --- |
|  | **Liver disease** | **Comparators** | **Liver disease** | **Comparators** | **Liver disease** | **Comparators** |  |  |
| **Overall** | 42 320 (100%) | 182 147 (100%) | 65 (0.15%) | 191 (0.10%) | 3.1 (2.4-3.9) | 2.1 (1.8-2.4) | 1.16 (0.87-1.55) | 1.08 (0.79-1.48) |
| Follow-up, months |  |  |  |  |  |  |  |  |
| 0-<1 | 42 320 (100%) | 182 147 (100%) | (0.00%) | -- (0.00%) | -- | -- | -- | -- |
| 1-<2 | 42 218 (99.8%) | 181 947 (99.9%) | 8 (0.02%) | 17 (0.01%) | 2.3 (0.7-3.9) | 1.1 (0.6-1.7) | 1.88 (0.80-4.38) | 1.70 (0.55-5.28) |
| 2-<3 | 42 080 (99.4%) | 181 752 (99.8%) | 29 (0.07%) | 85 (0.05%) | 8.3 (5.3-11.3) | 5.6 (4.4-6.8) | 1.15 (0.75-1.77) | 1.19 (0.72-1.95) |
| 3-<4 | 41 950 (99.1%) | 181 493 (99.6%) | 20 (0.05%) | 58 (0.03%) | 5.7 (3.2-8.2) | 3.8 (2.8-4.8) | 1.12 (0.67-1.89) | 1.11 (0.61-2.01) |
| 4-<5 | 41 850 (98.9%) | 181 293 (99.5%) | 8 (0.02%) | 28 (0.02%) | 2.3 (0.7-3.9) | 1.9 (1.2-2.5) | 1.05 (0.47-2.31) | 0.85 (0.33-2.21) |
| 5-<6 | 41 748 (98.6%) | 181 115 (99.4%) | (0.00%) | 3 (0.00%) | -- | 0.2 (0.0-0.4) | -- | -- |
| Sex |  |  |  |  |  |  |  |  |
| Females | 21 591 (51.0%) | 93 906 (51.6%) | 29 (0.13%) | 80 (0.09%) | 2.7 (1.7-3.7) | 1.7 (1.3-2.1) | 1.17 (0.76-1.81) | 1.11 (0.69-1.79)^Ψ^ |
| Males | 20 729 (49.0%) | 88 241 (48.4%) | 36 (0.17%) | 111 (0.13%) | 3.5 (2.4-4.7) | 2.5 (2.1-3.0) | 1.16 (0.79-1.70) | 1.07 (0.69-1.66) |
| Age at index date |  |  |  |  |  |  |  |  |
| <18 years | 2 769 (6.5%) | 12 877 (7.1%) | (0.00%) | -- (0.00%) | -- | -- | -- | -- |
| 18 - <40 years | 13 574 (32.1%) | 61 290 (33.6%) | 8 (0.06%) | 19 (0.03%) | 1.2 (0.4-2.0) | 0.6 (0.3-0.9) | 1.83 (0.80-4.20) | 0.44 (0.10-1.99)^Ψ^ |
| 40 - <60 years | 18 446 (43.6%) | 79 416 (43.6%) | 33 (0.18%) | 81 (0.10%) | 3.6 (2.4-4.9) | 2.1 (1.6-2.5) | 1.57 (1.04-2.37) | 1.77 (1.12-2.80) |
| ≥60 years | 7 531 (17.8%) | 28 564 (15.7%) | 24 (0.32%) | 91 (0.32%) | 6.6 (3.9-9.2) | 6.5 (5.2-7.8) | 0.75 (0.48-1.20) | 0.64 (0.38-1.09) |
| Index year |  |  |  |  |  |  |  |  |
| 1969-1989 | 2 966 (7.0%) | 11 276 (6.2%) | 6 (0.20%) | 14 (0.12%) | 4.1 (0.8-7.4) | 2.5 (1.2-3.8) | 1.02 (0.38-2.75) | 0.57 (0.17-1.89) |
| 1990-1999 | 11 722 (27.7%) | 47 935 (26.3%) | 21 (0.18%) | 57 (0.12%) | 3.6 (2.1-5.2) | 2.4 (1.8-3.0) | 1.19 (0.71-1.99) | 1.29 (0.75-2.22) |
| 2000-2009 | 15 979 (37.8%) | 69 263 (38.0%) | 21 (0.13%) | 68 (0.10%) | 2.7 (1.5-3.8) | 2.0 (1.5-2.5) | 1.10 (0.67-1.81) | 0.98 (0.53-1.80) |
| 2010-2017 | 11 653 (27.5%) | 53 673 (29.5%) | 17 (0.15%) | 52 (0.10%) | 3.0 (1.6-4.4) | 2.0 (1.4-2.5) | 1.26 (0.72-2.20) | 1.20 (0.60-2.38) |
| Age at start of follow-up |  |  |  |  |  |  |  |  |
| <18 years | 508 (1.2%) | 2 479 (1.4%) | -- (0.00%) | -- (0.00%) | -- | -- | -- | -- |
| 18 - <40 years | 4 105 (9.7%) | 19 274 (10.6%) | 1 (0.02%) | -- (0.00%) | 0.5 (0.0-1.5) | -- | -- | -- |
| 40 - <60 years | 13 567 (32.1%) | 62 844 (34.5%) | 10 (0.07%) | 15 (0.02%) | 1.5 (0.6-2.4) | 0.5 (0.2-0.7) | 3.15 (1.41-7.02) | 2.20 (0.67-7.28)^Ψ^ |
| ≥60 years | 24 140 (57.0%) | 97 550 (53.6%) | 54 (0.22%) | 176 (0.18%) | 4.6 (3.3-5.8) | 3.7 (3.1-4.2) | 1.02 (0.74-1.39) | 0.97 (0.69-1.37) |
| Country of birth |  |  |  |  |  |  |  |  |
| Nordic | 36 036 (85.2%) | 162 473 (89.2%) | 51 (0.14%) | 145 (0.09%) | 2.9 (2.1-3.7) | 1.8 (1.5-2.1) | 1.11 (0.80-1.55) | 1.05 (0.73-1.51) |
| Other | 6 284 (14.8%) | 19 670 (10.8%) | 14 (0.22%) | 46 (0.23%) | 4.5 (2.1-6.9) | 4.7 (3.4-6.1) | 2.39 (0.76-7.49) | 0.48 (0.04-5.09) |
| Level of education |  |  |  |  |  |  |  |  |
| ≤9 years | 9 467 (22.4%) | 33 283 (18.3%) | 21 (0.22%) | 66 (0.20%) | 4.5 (2.6-6.5) | 4.0 (3.1-5.0) | 1.23 (0.59-2.56) | 1.17 (0.51-2.68) |
| 10-12 years | 20 139 (47.6%) | 83 677 (45.9%) | 32 (0.16%) | 76 (0.09%) | 3.2 (2.1-4.3) | 1.8 (1.4-2.3) | 0.98 (0.59-1.63) | 1.21 (0.65-2.26) |
| >12 years | 12 567 (29.7%) | 64 460 (35.4%) | 11 (0.09%) | 46 (0.07%) | 1.8 (0.7-2.8) | 1.4 (1.0-1.9) | 1.53 (0.65-3.65) | 1.41 (0.46-4.33) |

Abbreviations: N., number; CI, confidence interval; PY, person-years; HR, hazard ratio

*Conditioned on matching set (age, sex, county, and calendar period);

**Conditioned on matching set and further adjusted for education, Nordic country of birth, and medical comorbidities at index date (cardiovascular disease, diabetes, chronic obstructive pulmonary disease, end-stage renal disease, alcohol use disorder, obesity/dyslipidemia, obstructive sleep apnea, cancer, psychiatric disease)

^Ψ^P-interaction values for sex, age at the index date and age at the start of follow-up = 0.90, 0.20 and 0.02, respectively

**Table S5.** Multivariate Cox proportional hazard regression models for the risk of hospital admission for COVID-19 and severe COVID-19

| **Covariate** | **Hospital admission for COVID-19** | | | **Severe COVID-19** | |
| --- | --- | --- | --- | --- | --- |
|  | **HR (95% CI)** | **P-value** | **HR (95% CI)** | | **P-value** |
|  |  |  |  | |  |
| **Exposure** |  |  |  | |  |
| Chronic Liver Disease | 1.36 (1.11-1.66) | 0.003 | 1.08 (0.79-1.48) | | 0.64 |
| Matched comparators (reference) | 1.00 | - | 1.00 | | - |
| **Level of education^3^** |  |  |  | |  |
| ≤9 years (reference) | 1.00 | - | 1.00 | | - |
| 10-12 years | 0.90 (0.71-1.15) | 0.41 | 0.72 (0.50-1.05) | | 0.09 |
| >12 years | 0.68 (0.52-0.89) | 0.005 | 0.55 (0.36-0.83) | | 0.004 |
| Missing | 1.20 (0.44-3.29) | 0.72 | 0.59 (0.15-2.36) | | 0.46 |
| **Country of birth** |  |  |  | |  |
| Nordic country | 0.30 (0.24-0.38) | <0.001 | 0.34 (0.23-0.51) | | <0.001 |
| Other (reference) | 1.00 | - | 1.00 | | - |
| **Disease history ever before index date^2^** |  |  |  | |  |
| Any cardiovascular disease | 1.27 (0.92-1.75) | 0.15 | 1.51 (0.93-2.43) | | 0.09 |
| Diabetes | 2.02 (1.33-3.07) | <0.001 | 2.19 (1.17-4.10) | | 0.01 |
| COPD | 0.95 (0.40-2.29) | 0.91 | 0.73 (0.22-2.47) | | 0.61 |
| End-stage renal disease | 5.84 (1.03-33.01) | 0.05 | 4.02 (0.21-76.36) | | 0.35 |
| Alcohol use disorder | 1.05 (0.62-1.79) | 0.85 | 1.92 (0.84-4.36) | | 0.12 |
| Obesity / Dyslipidemia | 1.49 (0.94-2.34) | 0.09 | 1.84 (1.02-3.35) | | 0.04 |
| Obstructive sleep apnea | 0.93 (0.39-2.22) | 0.86 | 0.71 (0.21-2.41) | | 0.58 |
| Cancer | 0.92 (0.61-1.37) | 0.67 | 0.78 (0.44-1.40) | | 0.41 |
| Psychiatric disease | 1.93 (1.39-2.69) | <0.001 | 1.94 (1.16-3.26) | | 0.01 |

Abbreviations: COPD, chronic obstructive pulmonary disease; HR, hazard ratio; CI, confidence interval

^2^The index date was defined as the date of liver biopsy confirming chronic liver disease (CLD), or the corresponding matching date among controls.

^3^Level of education was defined in 4 categories; among subjects with missing level of education, then the highest attained education level among parents was used.

**Table S6.** Characteristics of Patients with Chronic Liver Disease (CLD; n=42,008) and Propensity Score-Matched Population Controls (n=208,004), on December 31, 2016

| **Characteristic** | **Liver disease**  **(n=42,008)** | **Matched comparators**  **(n=208,004)** |
| --- | --- | --- |
| Females, no. (%) | 21 461 (51.1%) | 106 431 (51.2%) |
| Males, no (%) | 20 547 (48.9%) | 101 573 (48.8%) |
| Age at start of follow-up^1^ |  |  |
| Mean (SD) | 61.0 (15.9) | 61.1 (15.8) |
| Median (IQR) | 62.9 (51.9-72.9) | 62.9 (51.9-72.9) |
| Range, min-max | 3.1-99.7 | 3.2-99.9 |
| *Categories, no. (%)* |  |  |
| <18 years | 455 (1.1%) | 2 199 (1.1%) |
| 18 - <40 years | 4 042 (9.6%) | 19 929 (9.6%) |
| 40 - <60 years | 13 467 (32.1%) | 66 567 (32.0%) |
| ≥60 years | 24 044 (57.2%) | 119 309 (57.4%) |
| Country of birth, no (%) |  |  |
| Nordic country | 35 777 (85.2%) | 176 802 (85.0%) |
| Other | 6 231 (14.8%) | 31 196 (15.0%) |
| Missing | (0.0%) | 6 (0.0%) |
| Level of education^3^, no (%) |  |  |
| ≤9 years | 9 383 (22.3%) | 46 206 (22.2%) |
| 10-12 years | 19 966 (47.5%) | 100 603 (48.4%) |
| >12 years | 12 515 (29.8%) | 60 810 (29.2%) |
| Missing | 144 (0.3%) | 385 (0.2%) |
| Index year^2^ |  |  |
| 1969-1989 | 2 947 (7.0%) | 14 618 (7.0%) |
| 1990-1999 | 11 655 (27.7%) | 57 798 (27.8%) |
| 2000-2009 | 15 858 (37.7%) | 78 542 (37.8%) |
| 2010-2017 | 11 548 (27.5%) | 57 046 (27.4%) |
| Comorbidities ever before December 31, 2016^4^, no. (%) | |  |
| Any cardiovascular disease | 14 759 (35.1%) | 72 585 (34.9%) |
| Diabetes | 5 807 (13.8%) | 26 822 (12.9%) |
| Chronic obstructive pulmonary disease | 1 739 (4.1%) | 6 824 (3.3%) |
| End-stage renal disease | 397 (0.9%) | 1 223 (0.6%) |
| Alcohol use disorder | 4 212 (10.0%) | 20 910 (10.1%) |
| Alcohol liver disease | 1 209 (2.9%) | 672 (0.3%) |
| Obesity / Dyslipidemia | 6 097 (14.5%) | 28 405 (13.7%) |
| Obstructive sleep apnea | 1 945 (4.6%) | 8 244 (4.0%) |
| Cancer | 8 528 (20.3%) | 40 832 (19.6%) |
| Psychiatric disease | 10 448 (24.9%) | 52 568 (25.3%) |

Abbreviations: no., number; y, years; SD, standard deviation; IQR, interquartile range

^1^Start date of follow-up was defined as February 1, 2020 (see Methods).

^2^The index date was defined as the date of liver biopsy confirming chronic liver disease (CLD), or the corresponding matching date among controls.

^3^Level of education was defined in 4 categories; among subjects with missing level of education, then the highest attained education level among parents was used.

^4^Complete covariate data were available through December 31, 2016, as outlined in the Methods. For definitions of comorbidities, see the Supplementary Appendix.

**Table S7.** Risk of COVID-19 in Patients with Chronic Liver Disease (n=42,008) and Propensity Score-Matched Population Controls (n=208,004) from February 1 to July 31, 2020

| **Outcome** | **N events (%)** | | **Time at risk (years)** | | **Incidence rate (95% CI)**  **per 1000 PY** | | **Adjusted HR***  **(95%CI)** |
| --- | --- | --- | --- | --- | --- | --- | --- |
|  | **Liver disease** | **Comparators** | **Liver disease** | **Comparators** | **Liver disease** | **Comparators** |  |
|  |  |  |  |  |  |  |  |
| **Main outcomes** |  |  |  |  |  |  |  |
| Hospital admission | 160 (0.38%) | 656 (0.32%) | 20 634 | 102 461 | 7.8 (6.6-9.0) | 6.4 (5.9-6.9) | 1.20 (1.01-1.43) |
| Severe COVID-19 | 65 (0.15%) | 276 (0.13%) | 20 664 | 102 579 | 3.1 (2.4-3.9) | 2.7 (2.4-3.0) | 1.16 (0.88-1.52) |
|  |  |  |  |  |  |  |  |
| **Secondary outcomes** |  |  |  |  |  |  |  |
| Main outcomes combined | 181 (0.43%) | 756 (0.36%) | 20 634 | 102 460 | 8.8 (7.5-10.0) | 7.4 (6.9-7.9) | 1.18 (1.00-1.39) |
| All-cause mortality | 612 (1.46%) | 2 157 (1.04%) | 20 670 | 102 594 | 29.6 (27.3-32.0) | 21.0 (20.1-21.9) | 1.38 (1.26-1.51) |
| Any COVID-19 | 560 (1.33%) | 2 457 (1.18%) | 20 561 | 102 131 | 27.2 (25.0-29.5) | 24.1 (23.1-25.0) | 1.13 (1.03-1.24) |

Abbreviations: N, number; PY, person-years; HR, hazard ratio; CI, confidence interval

* Conditioned on matching set (age, sex, county, calendar period, education), Nordic country of birth and medical comorbidities up to December 31, 2016: (i.e. cardiovascular disease, diabetes, chronic obstructive pulmonary disease, end-stage renal disease, alcohol use disorder, obesity/dyslipidemia, obstructive sleep apnea, cancer, psychiatric disease)

**Table S8A.** Risk of COVID-19 Hospitalization Overall and by Subgroups in Patients with Chronic Liver Disease (n=42,008) and Propensity Score-Matched Population Controls (n=208,004) from February 1 to July 31, 2020.

| **Group** | **N (%)** | | **N events (%)** | | **Incidence rate (95% CI)**  **per 1000 PY** | | **Adjusted HR***  **(95%CI)** |
| --- | --- | --- | --- | --- | --- | --- | --- |
|  | **Liver disease** | **Comparators** | **Liver disease** | **Comparators** | **Liver disease** | **Comparators** |  |
| **Overall** | 42 008 (100.0%) | 208 004 (100.0%) | 160 (0.38%) | 656 (0.32%) | 7.8 (6.6-9.0) | 6.4 (5.9-6.9) | 1.20 (1.01-1.43) |
| Follow-up, months |  |  |  |  |  |  |  |
| 0-<1 | 42 008 (100.0%) | 208 004 (100.0%) | -- (0.00%) | -- (0.00%) | -- | -- | -- |
| 1-<2 | 41 907 (99.8%) | 207 689 (99.8%) | 27 (0.06%) | 113 (0.05%) | 7.7 (4.8-10.7) | 6.5 (5.3-7.7) | 1.18 (0.78-1.80) |
| 2-<3 | 41 752 (99.4%) | 207 214 (99.6%) | 68 (0.16%) | 277 (0.13%) | 19.6 (14.9-24.2) | 16.1 (14.2-18.0) | 1.21 (0.92-1.57) |
| 3-<4 | 41 575 (99.0%) | 206 472 (99.3%) | 49 (0.12%) | 149 (0.07%) | 14.2 (10.2-18.1) | 8.7 (7.3-10.1) | 1.62 (1.17-2.24) |
| 4-<5 | 41 440 (98.6%) | 206 012 (99.0%) | 13 (0.03%) | 104 (0.05%) | 3.8 (1.7-5.8) | 6.1 (4.9-7.2) | 0.61 (0.34-1.09) |
| 5-<6 | 41 334 (98.4%) | 205 606 (98.8%) | 3 (0.01%) | 13 (0.01%) | 0.9 (0.0-2.0) | 0.8 (0.4-1.2) | 1.09 (0.31-3.82) |
| Sex |  |  |  |  |  |  |  |
| Females | 21 461 (51.1%) | 106 431 (51.2%) | 60 (0.28%) | 279 (0.26%) | 5.7 (4.3-7.1) | 5.3 (4.7-5.9) | 1.05 (0.79-1.39) |
| Males | 20 547 (48.9%) | 101 573 (48.8%) | 100 (0.49%) | 377 (0.37%) | 9.9 (8.0-11.8) | 7.5 (6.8-8.3) | 1.31 (1.05-1.63) |
| Age at index date |  |  |  |  |  |  |  |
| <18 years | 2 669 (6.4%) | 13 043 (6.3%) | 2 (0.07%) | 8 (0.06%) | 1.5 (0.0-3.6) | 1.2 (0.4-2.1) | 1.25 (0.27-5.89) |
| 18 - <40 years | 13 470 (32.1%) | 66 605 (32.0%) | 37 (0.27%) | 144 (0.22%) | 5.6 (3.8-7.3) | 4.4 (3.7-5.1) | 1.25 (0.87-1.80) |
| 40 - <60 years | 18 361 (43.7%) | 91 058 (43.8%) | 85 (0.46%) | 303 (0.33%) | 9.4 (7.4-11.4) | 6.8 (6.0-7.5) | 1.41 (1.11-1.79) |
| ≥60 years | 7 508 (17.9%) | 37 298 (17.9%) | 36 (0.48%) | 201 (0.54%) | 9.9 (6.7-13.1) | 11.1 (9.5-12.6) | 0.88 (0.62-1.25) |
| Index year |  |  |  |  |  |  |  |
| 1969-1989 | 2 947 (7.0%) | 14 618 (7.0%) | 8 (0.27%) | 40 (0.27%) | 5.5 (1.7-9.4) | 5.6 (3.8-7.3) | 1.04 (0.49-2.23) |
| 1990-1999 | 11 655 (27.7%) | 57 798 (27.8%) | 50 (0.43%) | 214 (0.37%) | 8.7 (6.3-11.2) | 7.5 (6.5-8.5) | 1.14 (0.84-1.56) |
| 2000-2009 | 15 858 (37.7%) | 78 542 (37.8%) | 55 (0.35%) | 229 (0.29%) | 7.1 (5.2-8.9) | 5.9 (5.2-6.7) | 1.19 (0.89-1.60) |
| 2010-2017 | 11 548 (27.5%) | 57 046 (27.4%) | 47 (0.41%) | 173 (0.30%) | 8.3 (5.9-10.7) | 6.2 (5.2-7.1) | 1.31 (0.95-1.81) |
| Age at start of follow-up |  |  |  |  |  |  |  |
| <18 years | 455 (1.1%) | 2 199 (1.1%) | -- (0.00%) | -- (0.00%) | -- | -- | -- |
| 18 - <40 years | 4 042 (9.6%) | 19 929 (9.6%) | 7 (0.17%) | 15 (0.08%) | 3.5 (0.9-6.1) | 1.5 (0.8-2.3) | 2.30 (0.92-5.78) |
| 40 - <60 years | 13 467 (32.1%) | 66 567 (32.0%) | 47 (0.35%) | 146 (0.22%) | 7.1 (5.0-9.1) | 4.4 (3.7-5.2) | 1.58 (1.14-2.19) |
| ≥60 years | 24 044 (57.2%) | 119 309 (57.4%) | 106 (0.44%) | 495 (0.41%) | 9.0 (7.3-10.7) | 8.5 (7.7-9.2) | 1.05 (0.85-1.30) |
| Country of birth |  |  |  |  |  |  |  |
| Nordic | 35 777 (85.2%) | 176 802 (85.0%) | 115 (0.32%) | 457 (0.26%) | 6.5 (5.3-7.7) | 5.2 (4.8-5.7) | 1.21 (0.98-1.49) |
| Other | 6 231 (14.8%) | 31 196 (15.0%) | 45 (0.72%) | 199 (0.64%) | 14.7 (10.4-19.0) | 12.9 (11.1-14.7) | 1.13 (0.78-1.62) |
| Level of education |  |  |  |  |  |  |  |
| ≤9 years | 9 383 (22.3%) | 46 206 (22.2%) | 50 (0.53%) | 215 (0.47%) | 10.9 (7.9-13.9) | 9.5 (8.2-10.8) | 1.00 (0.72-1.41) |
| 10-12 years | 19 966 (47.5%) | 100 603 (48.4%) | 75 (0.38%) | 293 (0.29%) | 7.6 (5.9-9.4) | 5.9 (5.2-6.6) | 1.29 (0.99-1.68) |
| >12 years | 12 515 (29.8%) | 60 810 (29.2%) | 34 (0.27%) | 143 (0.24%) | 5.5 (3.7-7.4) | 4.8 (4.0-5.5) | 1.08 (0.71-1.62) |
| Cirrhosis |  |  |  |  |  |  |  |
| No Cirrhosis | 39,459 (93.9%) | 195 389 (93.9%) | 148 (0.38%) | 597 (0.31%) | 7.6 (6.4-8.9) | 6.2 (5.7-6.7) | 1.22 (1.02-1.46) |
| Cirrhosis | 2,549 (6.1%) | 12,615 (6.1%) | 12 (0.47%) | 59 (0.47%) | 9.6 (4.2-15.1) | 9.5 (7.1-11.9) | 0.99 (0.53-1.85) |
|  |  |  |  |  |  |  |  |

Abbreviations: N, number; PY, person-years; HR, hazard ratio; CI, confidence interval

*Conditioned on matching set (age, sex, county, calendar period, education), Nordic country of birth and medical comorbidities up to December 31, 2016: (i.e. cardiovascular disease, diabetes, chronic obstructive pulmonary disease, end-stage renal disease, alcohol use disorder, obesity/dyslipidemia, obstructive sleep apnea, cancer, psychiatric disease)

**Table S8B.** Risk of severe COVID-19 overall and by Subgroups in Patients with Chronic Liver Disease (n=42,008) and Propensity Score-Matched Population Controls (n=208,004) from February 1 to July 31, 2020.

| **Group** | **N (%)** | | **N events (%)** | | **Incidence rate (95% CI)**  **per 1000 PY** | | **Adjusted HR***  **(95%CI)** |
| --- | --- | --- | --- | --- | --- | --- | --- |
|  | **Liver disease** | **Comparators** | **Liver disease** | **Comparators** | **Liver disease** | **Comparators** |  |
| **Overall** | 42 008 (100.0%) | 208 004 (100.0%) | 65 (0.15%) | 276 (0.13%) | 3.1 (2.4-3.9) | 2.7 (2.4-3.0) | 1.16 (0.88-1.52) |
| Follow-up, months |  |  |  |  |  |  |  |
| 0-<1 | 42 008 (100.0%) | 208 004 (100.0%) | (0.00%) | (0.00%) | 0.0 (0.0-0.0) | 0.0 (0.0-0.0) | - |
| 1-<2 | 41 907 (99.8%) | 207 689 (99.8%) | 8 (0.02%) | 25 (0.01%) | 2.3 (0.7-3.9) | 1.4 (0.9-2.0) | 1.57 (0.71-3.47) |
| 2-<3 | 41 772 (99.4%) | 207 305 (99.7%) | 29 (0.07%) | 145 (0.07%) | 8.3 (5.3-11.4) | 8.4 (7.0-9.8) | 1.00 (0.67-1.49) |
| 3-<4 | 41 644 (99.1%) | 206 751 (99.4%) | 20 (0.05%) | 61 (0.03%) | 5.8 (3.2-8.3) | 3.5 (2.7-4.4) | 1.56 (0.94-2.59) |
| 4-<5 | 41 545 (98.9%) | 206 398 (99.2%) | 8 (0.02%) | 41 (0.02%) | 2.3 (0.7-3.9) | 2.4 (1.7-3.1) | 0.98 (0.46-2.10) |
| 5-<6 | 41 446 (98.7%) | 206 068 (99.1%) | (0.00%) | 4 (0.00%) | 0.0 (0.0-0.0) | 0.2 (0.0-0.5) | - |
| Sex |  |  |  |  |  |  |  |
| Females | 21 461 (51.1%) | 106 431 (51.2%) | 29 (0.14%) | 114 (0.11%) | 2.7 (1.7-3.7) | 2.2 (1.8-2.6) | 1.25 (0.83-1.89) |
| Males | 20 547 (48.9%) | 101 573 (48.8%) | 36 (0.18%) | 162 (0.16%) | 3.6 (2.4-4.7) | 3.2 (2.7-3.7) | 1.09 (0.76-1.57) |
| Age at index date |  |  |  |  |  |  |  |
| <18 years | 2 669 (6.4%) | 13 043 (6.3%) | (0.00%) | (0.00%) | 0.0 (0.0-0.0) | 0.0 (0.0-0.0) | - |
| 18 - <40 years | 13 470 (32.1%) | 66 605 (32.0%) | 8 (0.06%) | 34 (0.05%) | 1.2 (0.4-2.0) | 1.0 (0.7-1.4) | 1.14 (0.53-2.46) |
| 40 - <60 years | 18 361 (43.7%) | 91 058 (43.8%) | 33 (0.18%) | 100 (0.11%) | 3.7 (2.4-4.9) | 2.2 (1.8-2.7) | 1.65 (1.11-2.45) |
| ≥60 years | 7 508 (17.9%) | 37 298 (17.9%) | 24 (0.32%) | 142 (0.38%) | 6.6 (3.9-9.2) | 7.8 (6.5-9.1) | 0.85 (0.55-1.31) |
| Index year |  |  |  |  |  |  |  |
| 1969-1989 | 2 947 (7.0%) | 14 618 (7.0%) | 6 (0.20%) | 21 (0.14%) | 4.1 (0.8-7.5) | 2.9 (1.7-4.2) | 1.56 (0.62-3.92) |
| 1990-1999 | 11 655 (27.7%) | 57 798 (27.8%) | 21 (0.18%) | 90 (0.16%) | 3.7 (2.1-5.2) | 3.2 (2.5-3.8) | 1.14 (0.71-1.84) |
| 2000-2009 | 15 858 (37.7%) | 78 542 (37.8%) | 21 (0.13%) | 104 (0.13%) | 2.7 (1.5-3.8) | 2.7 (2.2-3.2) | 0.99 (0.62-1.59) |
| 2010-2017 | 11 548 (27.5%) | 57 046 (27.4%) | 17 (0.15%) | 61 (0.11%) | 3.0 (1.6-4.4) | 2.2 (1.6-2.7) | 1.33 (0.78-2.29) |
| Age at start of follow-up |  |  |  |  |  |  |  |
| <18 years | 455 (1.1%) | 2 199 (1.1%) | (0.00%) | (0.00%) | 0.0 (0.0-0.0) | 0.0 (0.0-0.0) | - |
| 18 - <40 years | 4 042 (9.6%) | 19 929 (9.6%) | 1 (0.02%) | 2 (0.01%) | 0.5 (0.0-1.5) | 0.2 (0.0-0.5) | 2.50 (0.23-27.57) |
| 40 - <60 years | 13 467 (32.1%) | 66 567 (32.0%) | 10 (0.07%) | 25 (0.04%) | 1.5 (0.6-2.4) | 0.8 (0.5-1.1) | 1.94 (0.93-4.04) |
| ≥60 years | 24 044 (57.2%) | 119 309 (57.4%) | 54 (0.22%) | 249 (0.21%) | 4.6 (3.4-5.8) | 4.2 (3.7-4.8) | 1.06 (0.79-1.43) |
| Country of birth |  |  |  |  |  |  |  |
| Nordic | 35 777 (85.2%) | 176 802 (85.0%) | 51 (0.14%) | 230 (0.13%) | 2.9 (2.1-3.7) | 2.6 (2.3-3.0) | 1.08 (0.80-1.47) |
| Other | 6 231 (14.8%) | 31 196 (15.0%) | 14 (0.22%) | 46 (0.15%) | 4.6 (2.2-6.9) | 3.0 (2.1-3.8) | 1.64 (0.79-3.38) |
| Level of education |  |  |  |  |  |  |  |
| ≤9 years | 9 383 (22.3%) | 46 206 (22.2%) | 21 (0.22%) | 102 (0.22%) | 4.6 (2.6-6.5) | 4.5 (3.6-5.4) | 0.85 (0.52-1.40) |
| 10-12 years | 19 966 (47.5%) | 100 603 (48.4%) | 32 (0.16%) | 127 (0.13%) | 3.3 (2.1-4.4) | 2.6 (2.1-3.0) | 1.29 (0.86-1.94) |
| >12 years | 12 515 (29.8%) | 60 810 (29.2%) | 11 (0.09%) | 44 (0.07%) | 1.8 (0.7-2.8) | 1.5 (1.0-1.9) | 1.14 (0.56-2.32) |
| Cirrhosis |  |  |  |  |  |  |  |
| No cirrhosis | 39,459 (93.9%) | 195 389 (93.9%) | 58 (0.15%) | 258 (0.13%) | 3.0 (2.2-3.8) | 2.7 (2.4-3.0) | 1.10 (0.83-1.46) |
| Cirrhosis | 2,549 (6.1%) | 12,615 (6.1%) | 7 (0.27%) | 18 (0.14%) | 5.6 (1.5-9.8) | 2.9 (1.6-4.2) | 2.11 (0.87-5.15) |
|  |  |  |  |  |  |  |  |

Abbreviations: N, number; PY, person-years; HR, hazard ratio; CI, confidence interval

*Conditioned on matching set (age, sex, county, calendar period, education, Nordic country of birth, and medical comorbidities at December 31, 2016 [cardiovascular disease, diabetes, COPD, end-stage renal disease, alcohol use disorder, obesity/dyslipidemia, obstructive sleep apnea, cancer, psychiatric disease])

**Table S9.** Baseline characteristics of Patients with Chronic Liver Disease (n=24,903) and Matched Full Sibling Comparators (n=45,169)

| **Characteristic** | **Liver disease**  **(n=24,903)** | **Siblings**  **(n=45,169)** |
| --- | --- | --- |
| Females, no. (%) | 12 436 (49.9%) | 22 830 (50.5%) |
| Males, no (%) | 12 467 (50.1%) | 22 339 (49.5%) |
| Age at start of follow-up^1^ |  |  |
| Mean (SD) | 59.3 (15.4) | 59.7 (15.4) |
| Median (IQR) | 61.6 (50.7-71.0) | 61.9 (51.3-71.2) |
| Range, min-max | 3.1-88.0 | 4.6-88.0 |
| *Categories, no. (%)* |  |  |
| <18 years | 316 (1.3%) | 519 (1.1%) |
| 18 - <40 years | 2 709 (10.9%) | 4 991 (11.0%) |
| 40 - <60 years | 8 371 (33.6%) | 14 858 (32.9%) |
| ≥60 years | 13 507 (54.2%) | 24 801 (54.9%) |
| Country of birth, no (%) |  |  |
| Nordic country | 24 097 (96.8%) | 43 262 (95.8%) |
| Other | 806 (3.2%) | 1 900 (4.2%) |
| Missing | (0.0%) | 7 (0.0%) |
| Level of education^3^, no (%) |  |  |
| ≤9 years | 5 035 (20.2%) | 9 534 (21.1%) |
| 10-12 years | 12 267 (49.3%) | 21 348 (47.3%) |
| >12 years | 7 594 (30.5%) | 13 160 (29.1%) |
| Missing | 7 (0.0%) | 1 127 (2.5%) |
| Index year^2^ |  |  |
| 1969-1989 | 1 802 (7.2%) | 3 245 (7.2%) |
| 1990-1999 | 7 036 (28.3%) | 12 726 (28.2%) |
| 2000-2009 | 9 199 (36.9%) | 16 761 (37.1%) |
| 2010-2017 | 6 866 (27.6%) | 12 437 (27.5%) |
| Comorbidities^4^ ever before index date^2^, no. (%) |  |  |
| Any cardiovascular disease | 3 253 (13.1%) | 3 036 (6.7%) |
| Diabetes | 1 249 (5.0%) | 965 (2.1%) |
| Chronic obstructive pulmonary disease | 271 (1.1%) | 264 (0.6%) |
| End-stage renal disease | 114 (0.5%) | 43 (0.1%) |
| Alcohol use disorder | 1 530 (6.1%) | 1 004 (2.2%) |
| Alcohol liver disease | 381 (1.5%) | 33 (0.1%) |
| Obesity / Dyslipidemia | 1 430 (5.7%) | 1 177 (2.6%) |
| Obstructive sleep apnea | 301 (1.2%) | 438 (1.0%) |
| Cancer | 3 380 (13.6%) | 1 188 (2.6%) |
| Psychiatric disease | 2 960 (11.9%) | 3 057 (6.8%) |
| Comorbidities ever before December 31, 2016^4^, no. (%) |  |  |
| Any cardiovascular disease | 8 293 (33.3%) | 9 833 (21.8%) |
| Diabetes | 3 214 (12.9%) | 2 862 (6.3%) |
| Chronic obstructive pulmonary disease | 985 (4.0%) | 988 (2.2%) |
| End-stage renal disease | 277 (1.1%) | 133 (0.3%) |
| Alcohol use disorder | 2 691 (10.8%) | 2 065 (4.6%) |
| Alcohol liver disease | 761 (3.1%) | 95 (0.2%) |
| Obesity / Dyslipidemia | 3 530 (14.2%) | 4 186 (9.3%) |
| Obstructive sleep apnea | 1 170 (4.7%) | 1 678 (3.7%) |
| Cancer | 5 014 (20.1%) | 3 572 (7.9%) |
| Psychiatric disease | 6 157 (24.7%) | 6 728 (14.9%) |

Abbreviations: CLD, chronic liver disease; no., number; SD, standard deviation; IQR, interquartile range

^1^Start date of follow-up was defined as February 1, 2020 (see Methods).

^2^The index date was defined as the date of liver biopsy confirming chronic liver disease (CLD), or the corresponding matching date among controls.

^3^Level of education was defined in 4 categories; among subjects with missing level of education, then the highest attained education level among parents was used.

^4^Complete covariate data were available through December 31, 2016, as outlined in the Methods. For definitions of comorbidities, see the Supplementary Appendix.

**Table S10.** Risk of COVID-19 Hospitalization in Patients with Chronic Liver Disease (n=24,903) and Full Siblings (n=45,169) from February 1 to July 31, 2020

| **Outcome** | **N events (%)** | | **Time at risk (years)** | | **Incidence rate (95% CI)**  **per 1000 PY** | | **HR***  **(95%CI)** | **Adjusted HR****  **(95%CI)** |
| --- | --- | --- | --- | --- | --- | --- | --- | --- |
|  | **Liver disease** | **Siblings** | **Liver disease** | **Siblings** | **Liver disease** | **Siblings** |  |  |
|  |  |  |  |  |  |  |  |  |
| **Main outcomes** |  |  |  |  |  |  |  |  |
| Hospital admission | 71 (0.29%) | 96 (0.21%) | 12 251 | 22 317 | 5.8 (4.4-7.1) | 4.3 (3.4-5.2) | 1.38 (1.00-1.89) | 1.42 (0.99-2.03) |
| Severe COVID-19 | 19 (0.08%) | 42 (0.09%) | 12 266 | 22 333 | 1.5 (0.9-2.2) | 1.9 (1.3-2.4) | 0.80 (0.45-1.40) | 1.09 (0.55-2.16) |
|  |  |  |  |  |  |  |  |  |
| **Secondary outcomes** |  |  |  |  |  |  |  |  |
| Main outcomes combined | 78 (0.31%) | 108 (0.24%) | 12 251 | 22 317 | 6.4 (5.0-7.8) | 4.8 (3.9-5.8) | 1.34 (0.99-1.82) | 1.38 (0.98-1.94) |
| All-cause mortality | 310 (1.24%) | 258 (0.57%) | 12 268 | 22 335 | 25.3 (22.5-28.1) | 11.6 (10.1-13.0) | 2.25 (1.89-2.67) | 1.88 (1.53-2.30) |
| Any COVID-19 | 271 (1.09%) | 465 (1.03%) | 12 214 | 22 252 | 22.2 (19.5-24.8) | 20.9 (19.0-22.8) | 1.06 (0.91-1.24) | 1.00 (0.84-1.18) |

Abbreviations: N, number; PY, person-years; HR, hazard ratio; CI, confidence interval

*Conditioned on matching set (family)

**Conditioned on matching set (family), with further adjustment for Nordic country of birth and medical comorbidities at the index date: (i.e. cardiovascular disease, diabetes, chronic obstructive pulmonary disease, end-stage renal disease, alcohol use disorder, obesity/dyslipidemia, obstructive sleep apnea, cancer, psychiatric disease)

**Figure S1. Flowchart of Chronic Liver Disease Patients and General Population Controls, Matched at Index Date^1^**

**
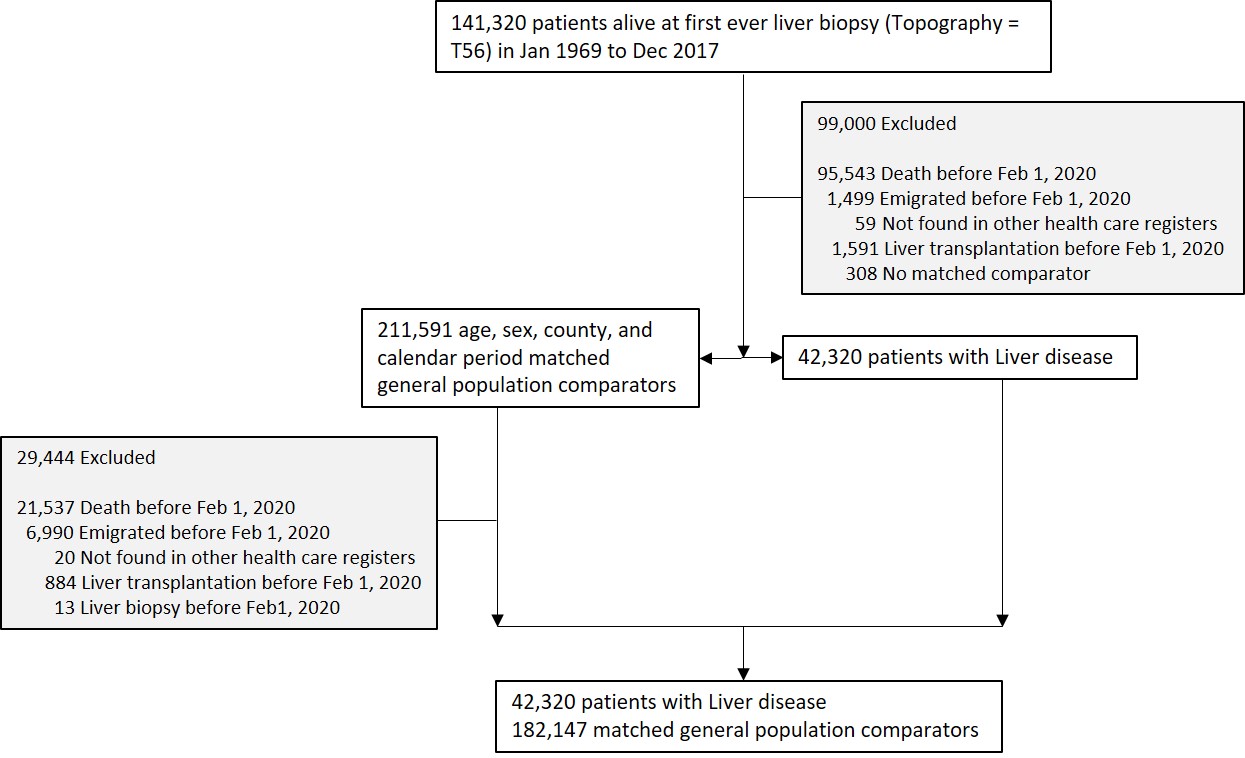
**

^1^Index date was the date of index liver biopsy (or the equivalent matching date, among population comparators

**Figure S2. Flowchart of CLD Patients and Population Controls, Propensity Score-Matched on December 31, 2016**

**
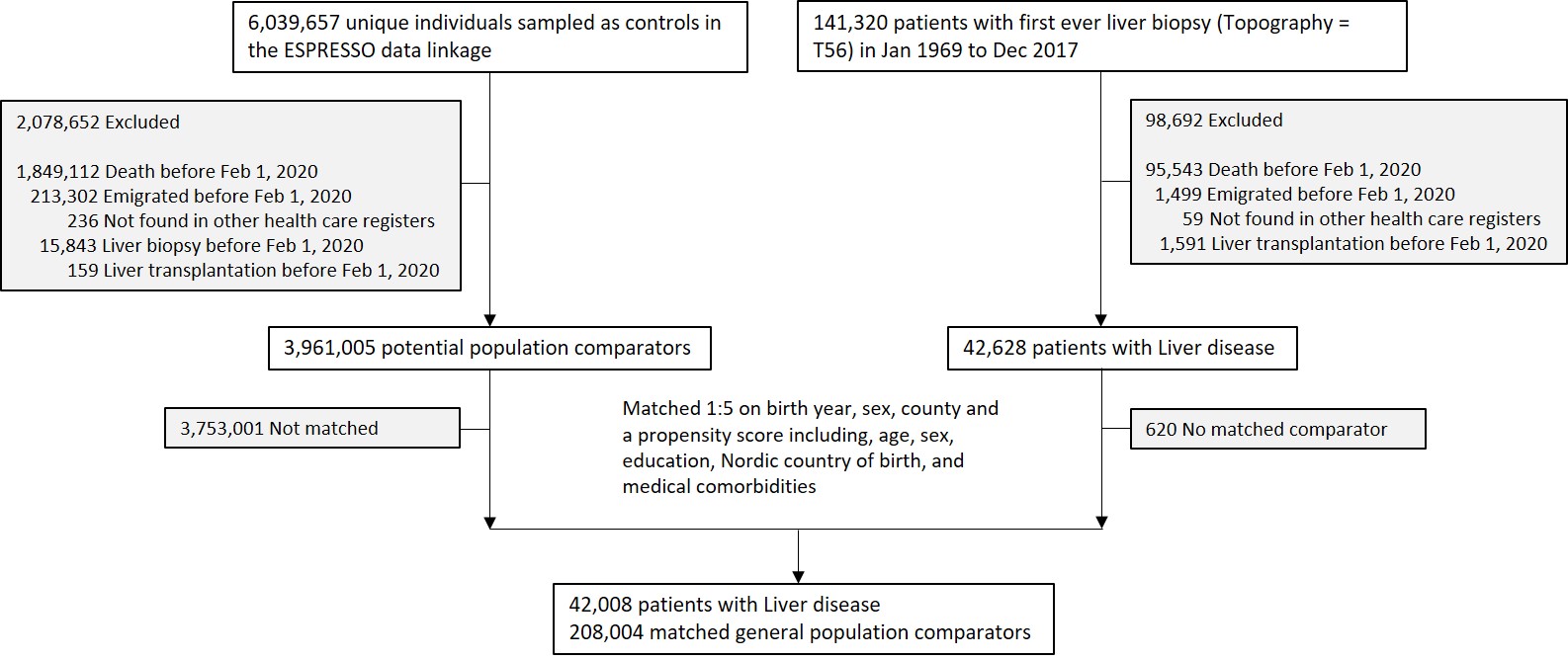
**

Abbreviations: CLD, chronic liver disease

**Figure S3.** Time to COVID-19 Hospitalization (Panel S3A) and the Development of Severe COVID-19 (Panel S3B), among Patients with Chronic Liver Disease (n=42,008) and Population Controls (n=208,004), Propensity Score-Matched on December 31, 2016^1^


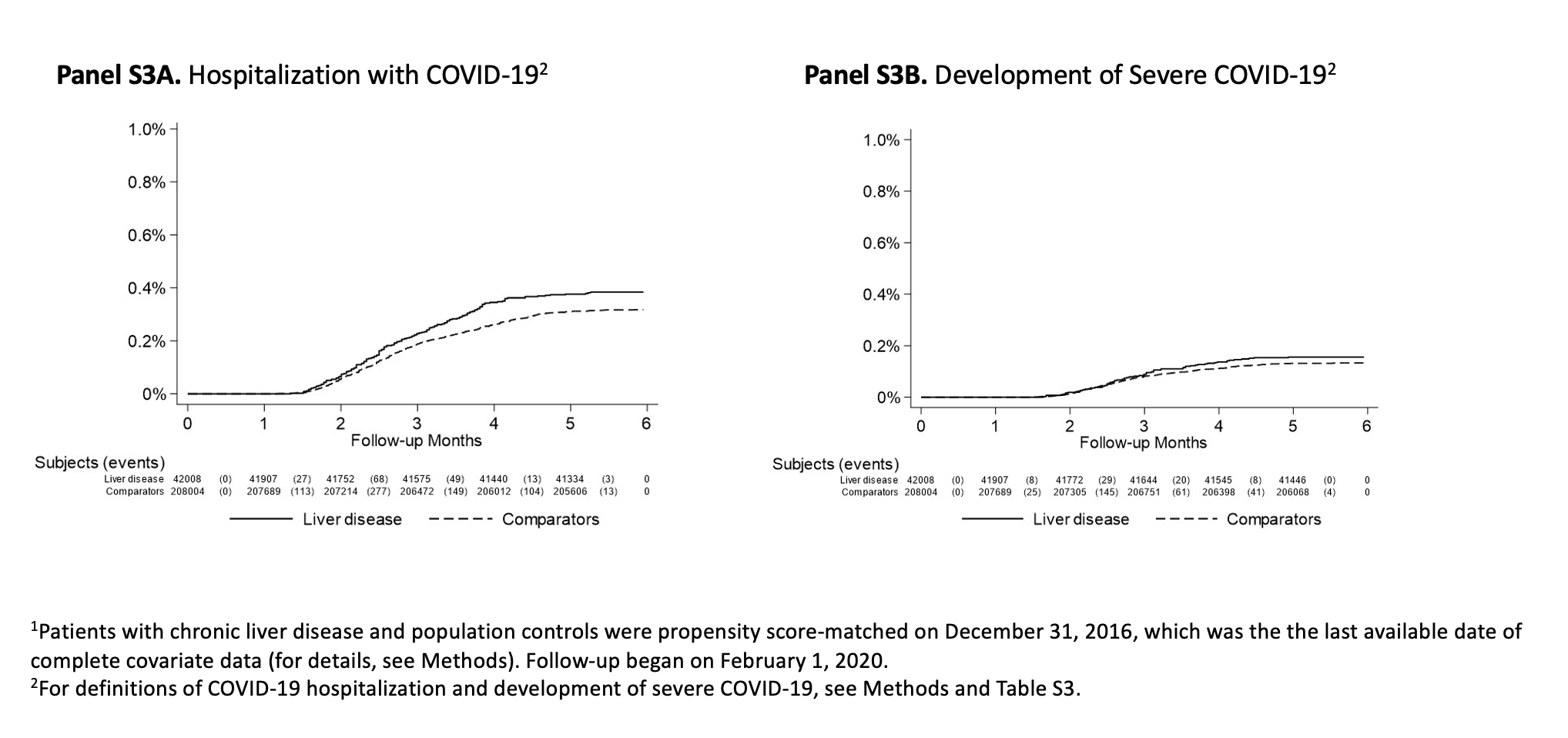


**Figure S4.** Time to COVID-19 Hospitalization (Panel S4A) and the Development of Severe COVID-19 (Panel S4B), among Patients with Chronic Liver Disease (n=24,903) and Full Sibling Comparators (n=45,169), Matched at the Index Date^1^

^
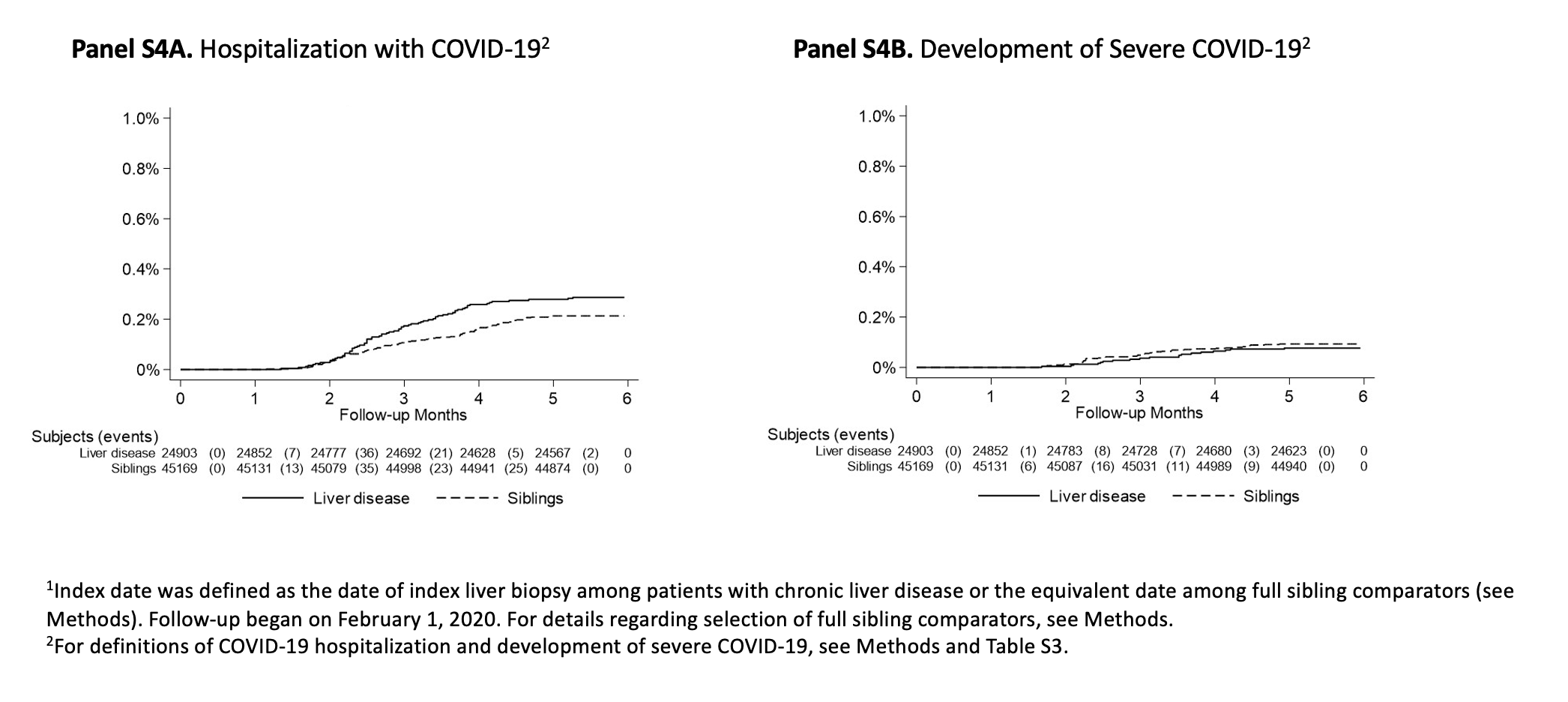
^

**Figure S5.** Time from any COVID-19 infection (Panel S5A) or Hospitalization with COVID-19 (Panel S5B) to All-Cause Mortality, among Patients with Chronic Liver Disease and Population Controls, Propensity Score-Matched on December 31, 2016^1^


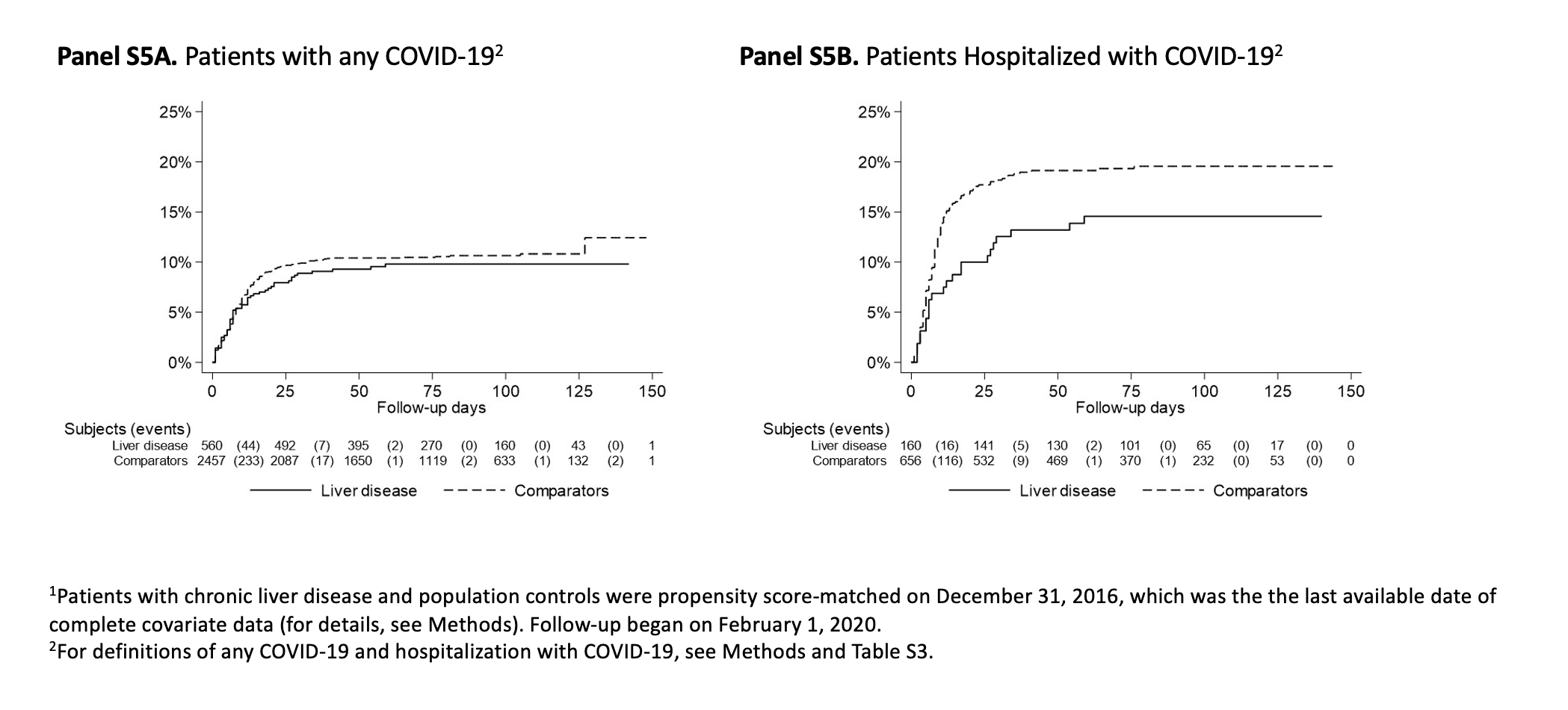

Supplement: Supplementary file 1 — Additional file 1: Definitions of chronic liver disease, baseline medical comorbidities, COVID-19 outcomes, study participants and risk estimates for COVID-19 in chronic liver disease. [file 12876_2021_2017_MOESM1_ESM.docx]
